# Molecular Landscapes and Models of Acute Erythroleukemia

**DOI:** 10.1097/HS9.0000000000000558

**Published:** 2021-04-21

**Authors:** Alexandre Fagnan, Maria-Riera Piqué-Borràs, Samantha Tauchmann, Thomas Mercher, Juerg Schwaller

**Affiliations:** 1 INSERM U1170, Equipe Labellisée Ligue Contre le Cancer, Gustave Roussy Institute, Université de Paris, Université Paris-Saclay, Villejuif, France; 2 University Children’s Hospital beider Basel (UKBB), Department of Biomedicine, University of Basel, Basel, Switzerland.

## Abstract

Malignancies of the erythroid lineage are rare but aggressive diseases. Notably, the first insights into their biology emerged over half a century ago from avian and murine tumor viruses-induced erythroleukemia models providing the rationale for several transgenic mouse models that unraveled the transforming potential of signaling effectors and transcription factors in the erythroid lineage. More recently, genetic roadmaps have fueled efforts to establish models that are based on the epigenomic lesions observed in patients with erythroid malignancies. These models, together with often unexpected erythroid phenotypes in genetically modified mice, provided further insights into the molecular mechanisms of disease initiation and maintenance. Here, we review how the increasing knowledge of human erythroleukemia genetics combined with those from various mouse models indicate that the pathogenesis of the disease is based on the interplay between signaling mutations, impaired TP53 function, and altered chromatin organization. These alterations lead to aberrant activity of erythroid transcriptional master regulators like GATA1, indicating that erythroleukemia will most likely require combinatorial targeting for efficient therapeutic interventions.

## Abstract 2

Supplemental Digital Content is available in the text.

## Introduction

First described in 1917 by Giovanni Di Guglielmo, acute erythroleukemia (AEL) accounts for 1%–5% of cases with acute myeloid leukemia (AML) and is generally associated with a poor prognosis.^1^ While most cases are identified in aged patients, AEL is occasionally also diagnosed in very young children. The cellular hallmark of AEL is impaired erythroid terminal differentiation and uncontrolled expansion of erythroid progenitor cells. AEL often presents with variable features that complicate the diagnosis which resulted in several changes in its classification over the years. The first French-American-British (FAB) system classified myeloid neoplasms with >30% leukemic blasts and ≥50% erythroid progenitor cells as AML-M6.^2^ Some AEL patients present with a heterogenous mixture of myeloid and erythroid features, while some less frequent cases present with >80% of erythroid progenitor cells considered as purely erythroid leukemia (PEL), respectively, called AML-M6a and AML-M6b in the WHO classification of 2008.^3^ Importantly, AEL patients may develop their disease de novo, but it frequently follows an antecedent myelodysplastic syndrome (MDS), myeloproliferative neoplasm (MPN), or therapeutic exposure to genotoxic agents. This suggests that the disease reflects a continuum of MDS and AML with erythroid hyperplasia. The clinicopathological overlap and the diagnostic difficulties to distinguish MDS from AEL led the WHO in 2016 to reclassify cases previously diagnosed as AEL into either MDS or PEL.^4^ Multiple studies have shown that leukemic blasts from AEL patients often carry complex karyotypes with frequent loss of chromosomes 5 and 7.^5^ Only recently, deep sequencing studies have revealed a more extensive genetic landscape of AEL beyond the most prevalent mutations. However, as outlined in the following, it is important to note that the first insights into the biology of AEL emerged already over half a century ago, when researchers (accidentally) developed erythroleukemia models while studying the in vivo activities of avian and murine tumor viruses. Mechanistic studies aiming to understand these unusual phenotypes initiated several more rationale erythroleukemia models. More recently, the established epigenomic AEL roadmap allowed to establish models that are based on the distinct AEL-associated lesions. These models together with completely unexpected erythroleukemia-like phenotypes in genetically modified mice provide a wide experimental platform to elucidate the molecular mechanisms of AEL initiation and maintenance. Here, we discuss how the increasing knowledge of AEL genetics combined with often unexpected findings from various erythroleukemia models allowed to generate new hypotheses on the molecular mechanisms driving AEL that may provide keys for future targeted therapeutic interventions.

## From complex karyotypes to epigenomic erythroleukemia landscapes

It has been recognized over 50 years ago that AEL leukemic blasts carry variable chromosomal abnormalities.^6,7^ Indeed, clonal chromosomal alterations are found in at least 75% of AEL patients and complex karyotypes were detected in at least 50% of patients.^8,9^ Complex or hypodiploid karyotypes were seen in at least 50% of cases with entire or partial monosomies of chromosome 5 and 7 being the most frequent.^10^

**Figure 1.**
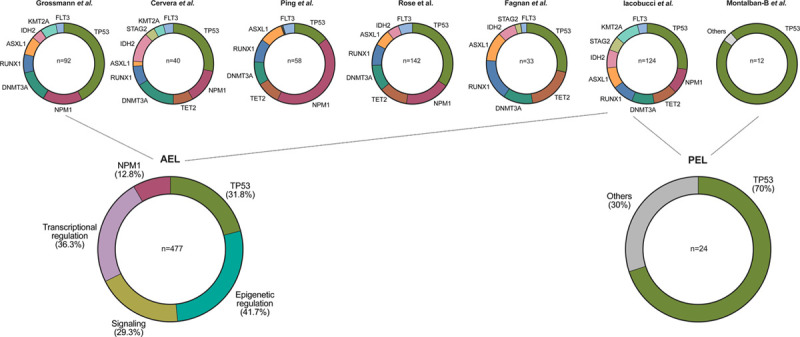
**Genetic landscape of human AEL.** Schematic representation of the most prevalent genetic lesions reported in human AEL patients.^14,15,17,19,21^ Alterations of the TP53 tumor suppressor were found in ≥30% of AEL and in the majority PEL patients. Note: only the studies by Iacobucci et al and Montalban-Bravo et al included patients with leukemia diagnosed as PEL (according to the WHO 2016 classification). AEL = acute erythroleukemia; PEL = purely erythroid leukemia.

Improved technologies have allowed the first targeted and then genome-wide sequencing of AEL patients samples, revealing recurrently mutated genes. Considering all published genetic studies, *TP53* is the most frequently mutated gene, identified in about 30% of the patients, associated with complex karyotype and poor outcome. Notably, *TP53* mutations have been identified in almost all patients with pure erythroid leukemia (PEL), thus representing a molecular hallmark of human erythroleukemia.^11–13^ The other recurrent mutations target *NPM1*, epigenetic regulators (including *TET2* and *ASXL1* loss of function mutations, *DNMT3A*^*R882*^ or *IDH2*^*R140*^), intermediates of signaling pathways as well as key hematopoietic transcription factors, which were also frequently identified in other AML subtypes.^14–21^ Signaling mutations have been reported in 25%–50% of AEL patients, including recurrent activating mutations of the JAK-STAT signaling (*JAK2*^*V617F*^, *FLT3*^*ITD*^, or *EPOR*) or the RAS (*NRAS*, *KRAS*, *PTPN11*, or *NF1*) pathways and are mostly associated with additional mutations such as *TP53* or *NPM1*. However, no single highly recurrent gene mutation has been reported to date supporting a high heterogeneity in the type of signaling mutation associated with AEL (Figure 1; Supplemental Digital Table 1, http://links.lww.com/HS/A149).

As the disease frequently develops secondary to MPN or MDS, known driver mutations of these disorders like the *BCR-ABL* fusion gene and activating mutations of *JAK2* are often associated with AEL.^10,21,22^ Notably, cytogenetic analysis of 75 AEL patients, 4 of 7 cases of pure erythroid leukemia were associated with a BCR-ABL fusion.^23^ Single BCR-ABL-positive AML-M6 cases were reported to enter into long-term remission upon treatment with the imatinib tyrosine kinase inhibitor.^24^ However, some cases of secondary AEL following a *JAK2*^*V617F*+^ MPN were reported to lack the JAK2 mutation in AEL blasts.^25^ Of note, several of the human erythroleukemia cell lines carry one of these tyrosine kinase mutations (eg, K562: *BCR-ABL*, HEL: *JAK2*^*V617F*^) suggesting that proliferative signals are essential to maintain these cells in vitro.^26^ Interestingly, Iacobucci et al^19^ also highlighted age-related differences in the mutational profiles of AEL patients associated with distinct prognosis, including higher prevalence of *TP53* mutation in >60-year-old patients and higher representation of NPM1, TET2, or DNMT3A in 20- to 59-year-old patients.

The concept of clonal hematopoiesis of indeterminate potential (CHIP) is now well established with the observation that several mutations are particularly associated with CHIP and can predispose to the development of hematopoietic malignancies, as well as other human diseases.^27,28^ Increasing evidence suggests that AEL represents the evolution of a continuum between normal hematopoiesis, CHIP, myeloid neoplasm, and acute leukemia. Indeed, recurrent mutations identified in CHIP including *TP53*, *DNMT3A*, and *TET2* are also frequently found in human erythroleukemia. Genetic analyses of AEL shows that several mutations often present with low-allelic frequency (eg, *ASXL1*, *PTPN11*, and *WT1*) evoking their post-MPN, -MDS, -CHIP origin. In contrast, *TP53* mutations in AEL are characterized by a high-allelic frequency in leukemic cells (often with evidence of bi-allelic inactivation) and are also often identified in other cell populations than the leukemic cells (ie, T-cell compartment), supporting the idea that they arise at an early time point during disease development in an immature multipotent hematopoietic progenitor. Together, these results support an early acquisition of genetic lesions (including those associated with CHIP) and acquisition of additional sometimes subclonal alterations compatible with the emergence of AEL directly or indirectly from a CHIP situation. However, this hypothesis will need to be formally proven.

Although rare, AEL can affect pediatric patients. Interestingly, some recurrent translocations have been described in pediatric AEL, which so far have never been associated with other AML forms including t(1;16)(p31;q24) and t(11;20)(p11;q11) leading to expression of *NFIA-ETO2* and *ZMYND8-RELA* fusions, respectively.^29–33^ Very recently, a novel t(1;8)(p31;q21) translocation leading to the expression of an *NFIA-ETO* fusion was described in an infant with PEL presenting as erythroblastic sarcoma.^34^ Fusions targeting the nucleopore components like NUP98 or NUP214, like *NUP98-NSD1*, *NUP98-KDM5A*, or *DEK-NUP214* described in other AML types have also been found in pediatric AEL.^35^ In addition, several less frequent fusions were identified, involving erythroid-associated factors (*MYB-GATA1* and *APLP2-EPOR*), epigenetic factors (*ZEB1-KDM4C* and *SMARCA4-CBS*), or signaling pathways like *ASNS-PTPN1*, *SRC-VWC2*, *RUNX2-STAT3* and *PRKAR2B-PIK3C*, or *PCM1-JAK2*.^15,19^ Of note, most of these fusions are individually very rare, suggesting that many different genetic alterations can lead to childhood AEL. Taken together, these studies proposed a molecular classification of AEL patients according to genetic and transcriptomic landscapes that could be associated with age at diagnosis and distinct clinical outcomes.

## From tumor viruses to rationale erythroleukemia models

**Figure 2.**
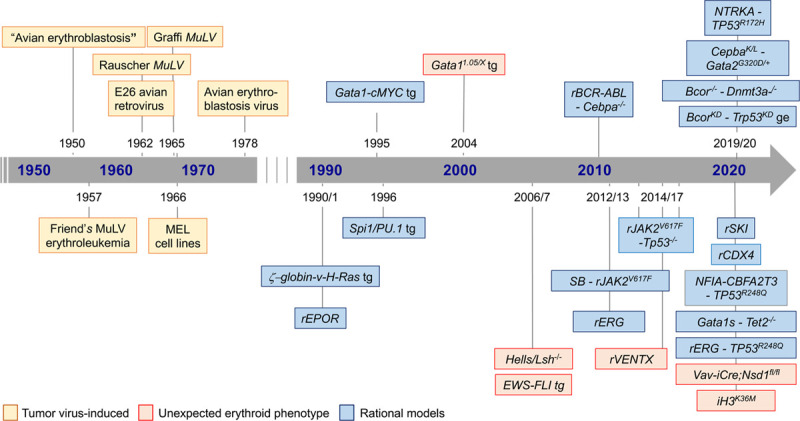
**Chronology of AEL mouse models.** Schematic timeline of tumor virus-induced, unexpected, and rational erythroleukemia mouse models. r = viral overexpression; tg = transgenic; ge = genome editing; kd = knock-down; MuLV = murine leukemia virus; AEL = acute erythroleukemia.

The first erythroleukemia models emerged over half a century ago mostly from phenotypes induced by tumor viruses. These models set the stage for more rational models exploring the transforming potential of signaling mediators and transcription factors in cells of the erythroid lineage. In addition to more recent models that explored the transforming activity of genetic alterations found in AEL patients, unexpected erythroleukemia phenotypes in genetically modified mice provided insight into epigenetic regulation of erythroid differentiation (Figure 2).

### Tumor virus-induced erythroleukemia models

#### Avian leukemia viruses

The first hematopoietic malignancies-inducing oncogenes have been discovered by studying the in vivo activity of avian viruses. The avian erythroblastosis retrovirus (AEV), encoding for viral oncogenic variants *v-ErbA* and *v-ErbB* of cellular genes (respectively, the thyroid hormone receptor alpha [TRα] and a mutated epithelial growth receptor) was found to induce fatal erythroleukemia in young chicken. AEV blocks terminal differentiation of committed erythroid progenitor cells. Further functional studies led to the hypothesis that *v-ErbA* cooperates with activated cellular stem cell factor receptor Kit and *v-ErbB* to efficiently arrest terminal erythroid maturation.^36,37^ Molecular studies delineated a more general mechanism of oncogenesis based on the inability of altered nuclear receptors to efficiently respond to physiological concentrations of ligands, which was also shown to be the driving force of other AML forms such as acute promyelocytic leukemia mediated by retinoid acid receptor alpha (RARA) fusion proteins.^38^

The E26 avian retrovirus, which induces massive erythroblastosis in newborn chicken, encodes for a fusion between a portion of the viral *gag* sequences to truncated mutated forms of the transcription factors MYB and ETS1.^39^ Functional studies revealed that *v-ets* is required for the E26-mediated block erythroid differentiation. Interestingly, the *Myb-Ets* fusion protein seems to inhibit *v-ErbA* and RARA, indicating overlapping pathways of malignant transformation by E26 and AEV.^40,41^

In 1957, Friend^42^ reported that intraperitoneal injection of cell-free extracts prepared from ascites of mice inoculated with Ehrlich’s carcinoma cells induced a leukemia-like disease. Electron microscopic analysis indicated that Ehrlich’s cells, derived from a spontaneous mouse mammary adenocarcinoma, contained particles similar to what has been seen in virus-infected cells.^42^ Intraperitoneal injections of spleen cell suspensions or filtrates into Swiss albino mice resulted in signs of disease in >80% of the recipients. Affected mice had significant infiltrations in hematopoietic organs by cells that looked like erythroid progenitor cells. Subsequent studies suggested that erythropoietin (EPO)-sensitive erythroid progenitors and in particular late burst forming unit or colony forming unit-erythroid (CFU-E) are the targets of the Friend leukemia virus. Permanent cell lines could be established called Friend tumor cells or murine erythroleukemia cells (MELs). Notably, the observation that particular chemicals (eg, dimethyl sulfoxide [DMSO]) are able to induce partial terminal erythroid differentiation made these cells one of the most widely used in vitro platform to study erythroid maturation.^43^ Similar to Friend’s, other viruses like the Rauscher or Graffi MuLV were shown to induce an erythroblastosis that phenocopied human erythroleukemia in mice.^44,45^

Friend virus contains 2 components, the replication-competent Friend murine leukemia virus acting as a helper for the replication-defective spleen focus forming virus (SFFV), which is the erythroblastosis-inducing component. The pathogenic activity is mediated by an env-derived viral 55 kDa glycoprotein (gp55) that directly interacts with and activates the EPO receptor (EPOR) promoting EPO-independent proliferation and differentiation. Recruitment of a cellular receptor tyrosine kinase receptor stk/RON by gp55 results in activation of downstream signaling effectors including signal activators of transcription (STATs), PI3K/AKT, or MAP kinases.^46^ Proviral integration cloning revealed that Friend virus integrated almost exclusively in a site called *SFFV-proviral integration site-1* (*Spi-1*), which resulted in transcriptional activation of the *Spi-1-1* gene locus by the viral LTR enhancers and in overexpression of the *Spi-1* mRNA.^47^

### Rationale erythroleukemia mouse models

#### Erythroleukemia by overexpression of a *Spi-1* transgene

To model the biological activity of aberrant *Spi-1* expression, Françoise Moreau-Gachelin and co-workers established a transgenic mouse model in which a *Spi-1* mini-gene was expressed under the control of the *SFFV-LTR* (Table 1). During an observation period of 12 months, 50% of *Spi-1* transgenic mice developed hepatosplenomegaly with extensive erythroblast infiltration and occasional tumor cells on the peripheral blood smears. Cells from diseased mice could be grown ex vivo as EPO-dependent cell lines but did not induce the disease upon transplantation, indicating that ectopic *Spi-1* expression blocks erythroid differentiation but does not overcome growth factor requirement for survival.^50^ Under hypertransfusion stress, tumor cells (referred as “HS-2 cells”) emerged that were able to proliferate independent of EPO and induce the disease in immunodeficient mice. HS-2 cells carried mutations of the Kit receptor tyrosine kinase leading to constitutive activation of PI3K/AKT and MAPK signaling,^58^ representing a good example of acquired mutation in signaling molecules as a cooperative mechanism contributing to differentiation blockage in cancer. Notably, erythroblasts from the late stage FLV-induced disease harbored allelic losses or missense *Trp53* mutations.^59^ Loss of *Trp53* alleles also increased penetrance and reduced the latency of erythroleukemia in *Spi-1* transgenic mice.^60^

**Table 1.** Rational Genetic AEL Mouse Models

Year	Gene	Model	Phenotype	Major Findings	Surface Markers on Leukemic Cells	References
1990	*EPOR*	*SFFV* ORF transduction.	Erythroleukemia	Polycythemia, splenomegaly	n.d.	^48^
1995	*c-MYC*	Transgene under control of *Gata1* enhancer/promoter	Early onset erythroleukemia, mice died before reaching sexual maturity	Splenomegaly, erythroid progenitors in peripheral blood, tumor cell infiltration, severe anemia, and moderate thrombocytopenia	n.d.	^49^
1996	*Spi-1*	Classical transgene (“mini gene”)	50% homozygous mice developed a multistep erythroleukemia within 1.5 to 6 mo of birth. Transplantable into nude recipients	Hepatosplenomegaly with erythroblast infiltration	Increase Ter119^+^, reduced B220^+^, CD4^+^, CD8^+^, Mac1^+^, Gr1^+^, Sca1^+^	^50^
1998	*H-Ras*	Transgene under control of *Zeta-globin* enhancer/promoter mouse	Mesenchymal and epithelial neoplasms, <5% erythroleukemia	Mesenchymal and epithelial neoplasms, <5% showed hepatosplenomegaly with erythroblast infiltration	Ter119^+^	^51^
2004	*GATA1*^*1.05/x*^	Transgenic mouse, insertion of *Neo* cassette before *Gata1*-GIE region	50% of GATA1^*1.05/x*^ mice developed disease: 2 phenotypes: myeloid disease after 143 d and lymphoid disease after median latency of 387 d. Transplantable into nude mice	Anemia, thrombocytopenia, erythroblasts, and megakaryocytes in spleens of mice with myeloid disease	Myeloid disease: Kit^+^, CD71^+^, Ter119^−/dull^, CD19	^52^
					Lymphoid disease: Kit^−^ Sca1^+^/CD43^+^/ CD19^+^	
2007	*EWS-FLI*	Inducible transgene in *Rosa26*, activated by *Mx1-iCre* (pIpC)	Rapid, highly penetrant (90%–100%) erythroleukemia (+pIpC: 19 d, −pIpC: 95 d). Transplantable in 19/26 sublethally irradiated wild-type and 7/7 NOD/SCID recipients	Anemia, peripheral blood blasts, no thrombocytopenia, hepatosplenomegaly with infiltration of tumor cells	Kit^+^, CD43^+^, CD71^+^. Many cells Gata1^+^ with increased c-Myc expression	^53^
2012	*ERG*	*MSCV* viral overexpression and BM reconstitution	Erythro-megakaryoblastic leukemia, T-cell acute lymphoblastic leukemia. Transplantable	Hematopoietic malignancies affecting erythroid, megakaryocytic, and T-cell lineage	CD71^+^/Ter119^+^, CD4^+^/CD8^+^	^54^
2012	*ERG*	*MSCV* viral overexpression and BM reconstitution	Lymphoid leukemia, erythroid-megakaryocytic leukemia. Transplantable (30/32)	Accumulation of immature erythroblasts in vivo, cell clones exhibited both erythroid and megakaryocytic differentiation in vitro	CD4^+^/CD8^+^	^55^
					CD71^+^/Ter119^+^, CD71^+^/CD41^+^	
2016	*VENTX*	*MSCV* viral overexpression and BM reconstitution	Erythroleukemia. Transplantable (13/19)	Infiltrations of BM and spleen by erythroblasts after long latency	Primary: Mac-1^+^, Mac-1/Gr-1^+^, Kit^low^, CD71^+^, Ter119^−^	^56^
					Secondary: Ter119^+^, partly CD71^+^	
2019	*CDX4*	*MSCV* viral overexpression and BM reconstitution	Erythroleukemia-like diseases after a long latency. Transplantable	Anemia, splenomegaly, infiltration of erythroid progenitors. Tumor cells expressed low levels of Gata1	CD71^+^/Ter119^+/−^	^57^
2020	*SKI*	*MSCV* viral overexpression and BM reconstitution	Leukemia of erythroid and myeloid phenotype	Pan-cytopenia associated with accumulation of erythroid and myeloid progenitors in BM, spleen and liver	CD71^+^/Ter119^+/−^	^21^
					CD11b^+^/Gr-1^+^	

AEL = acute erythroleukemia; BM = bone marrow; MSCV = murine stem cell virus.

#### Erythroleukemia by constitutive EPOR activation

SFFV-encoded gp55 glycoprotein binds and activates the EPOR bypassing the cellular requirement for EPO and supporting proliferation and survival of infected erythroid cells.^61^ To address the leukemogenic potential of a constitutively active EPOR, Longmore and Lodish^48^ generated an SFFV in which they replaced *gp55* with a mutated *EPOR*. Injection of this modified SSFV-induced polycythemia and splenomegaly in mice.^48^ Transfer of growth-factor-independent erythroblasts isolated from the spleens of these mice rapidly induced an erythroleukemia-like disease in the recipients. Of note, tumor cells did neither secreted a pathogenic virus nor did they integrated into *Spi-1*, but they carried inactivating *Trp53* rearrangements. Aberrant EPO expression was also found in serially propagated FLV erythroleukemia cell lines, due to genomic rearrangements independent of retroviral integration.^62^

#### Erythroleukemia by targeted expression of master oncogenes

In vitro studies showed that DMSO-induced MEL cell erythroid differentiation is associated with reduced expression of the *MYC* proto-oncogene and that its overexpression inhibited differentiation.^63,64^ To study the in vivo transforming potential of *MYC* in the erythroid lineage, Phil Leder and co-workers used regulatory sequences potentially controlling the expression of the erythroid master regulator GATA1 in transgenic mice.^49^ Diseased mice presented with splenomegaly with significant tumor cell infiltration and erythroid progenitors in the peripheral blood. Tumor cells showed clonogenic activity in methylcellulose (MC) without growth factors and induced the same disease phenotype when transplanted. Furthermore, tumor cells expressed erythroid genes (*EPOR*, *globin*), but, unlike MEL cells, exposure to DMSO did not induce terminal differentiation. These observations suggest that aberrant *MYC* activation at a particular vulnerable phase of erythroid differentiation is most likely sufficient to induce erythroleukemia.

In addition to *MYC*, increased levels of the *H-Ras* and *K-Ras* oncogenes were found in Friend’s murine erythroleukemia.^65^ Leder and co-workers established another series of transgenic mice in which the embryonic alpha-like zeta-globin gene was driving expression of an activated *H-Ras* oncogene. Unexpectedly, these transgenic mice (“Tg.AC”) developed multiple mesenchymal and epithelial neoplasms and only few mice (<5%) showed hepatosplenomegaly with erythroblast infiltration.^51^ Impaired EPO-induced differentiation of an FLV-induced erythroleukemia cell line (SKT6) by a constitutively active *H-Ras*^*G12V*^ mutant also suggested that aberrant RAS activation can enhance erythroid transformation.^66^

#### Erythroleukemia by aberrant activity of erythroid transcriptional regulators

##### GATA binding protein 1

The *GATA1* gene on chromosome X encodes for a zinc-finger transcription factor expressed in erythroid, megakaryocytic, eosinophilic, and mast hematopoietic cells as well as in Sertoli cells of the testes.^67^ As the generation of germline *Gata1*-null alleles resulted in embryonic lethality, Yamamoto and colleagues aimed to alter *Gata1* expression by inserting a *neomycin* selection cassette in the promoter region between the so-called double GATA sequence and the erythroid-specific exon 1 (IE). Whereas hemizygous mutant male embryos died in utero due to impaired primitive erythropoiesis, heterozygous female mice survived due to random inactivation of the X-chromosome. As these mice expressed about 5% of *Gata1* transcripts, this targeted mutation was referred as the *Gata1*^*1.05*^ allele. While the decreased number of erythroid cells and CFU-E formation was observed in fetal livers, accumulation of primitive erythroid progenitors was observed as early as E9.5 in mutant mice.^68^ Heterozygous *Gata1*^*1.05/X*^ female mice developed signs of distress at the age of 5 months presenting with anemia, thrombocytopenia with massive accumulation of erythroblasts and megakaryocytes in their spleens.^69^ Detailed analysis of a larger cohort of heterozygous female *Gata1*^*1.05/X*^ mice revealed a high incidence (~50% penetrance) of leukemia composed of either Kit^+^ erythroid blasts (starting at 143 d) or CD19^+^ lymphoid blasts (starting at 387 d). Tumor cells were Kit^+^/CD71^+^/Ter119^−/dull^/CD19^−^ proerythroblasts. Tracking of the cells with a GATA1-controlled fluorescent reporter suggested that immature erythroid cells were already expanding in the hematopoietic organs of *GATA-1*^*1.05/X*^ mice at the late embryonic stages.^52^

Several models indicate that GATA1 activity tightly controls the balance between proliferative erythroid progenitors and maturing cells. First, in the *Gata1*^*1.05/X*^ model, leukemia development is completely abolished by transgenic expression of wild-type *Gata1*. A cell line (“GAK-14”) was established from *Gata-1*^*1.05/X*^ diseased mice that maintained an immature erythroblastic phenotype (CD71^+^/Kit^+^/Ter119^−^) when grown on OP9 stroma cells in the presence of EPO and stem cell factor. Retroviral overexpression of *Gata1* resulted in GAK-14 differentiation into mature erythroid cells when cultured on fetal liver-derived stroma cells.^70^ Similarly, through expression of the apoptosis inhibitor BCL2 into *Gata1-deficient* embryonic stem cells followed by in vitro erythroid differentiation, Weiss et al^71^ generated a stable erythroblastic cell line (“G1E”). Exogenous Gata1 expression in G1E cells restored erythroid maturation^71^ and allowed to functionally dissect Gata1 critical domains, posttranslational modifications and target genes.^72–74^ Collectively, the observations suggested that impaired GATA1 activity is an important feature for induction and most likely also maintenance of transformed murine erythroid progenitor cells.^75^

##### ETS transcription factor ERG

ERG is a member of the E26 transformation-specific family of transcription factors that contain a highly conserved ETS DNA binding domain that interacts together with other transcription factors to enhancer elements.^76^ Functional studies in mice revealed that *ERG* expression promotes HSC maintenance but also controls erythromegakaryocytic differentiation.^77–79^ ERG was found to bind together with GATA1 to regulatory elements of key hematopoietic transcription factors like SCL/TAL1.^80^ The *ERG* gene is targeted by several chromosomal translocations associated with AML but also solid tumors.^81^ High *ERG* expression levels have been associated with poor prognosis in cytogenetically normal AML.^82^ In addition, increased *ERG* gene dosage seems to cooperate with the N-terminal GATA1 mutation (GATA1s) in the transient myeloproliferative disorder associated with Down’s syndrome.^83^ *ERG* is not only highly expressed in trisomy 21-related but also in sporadic cases of acute megakaryoblastic leukemia (AMKL). Increased *ERG* expression was shown to promote in vitro megakaryopoiesis and synergize with GATA1 to immortalize hematopoietic progenitor cells.^84^

Several groups explored the oncogenic potential of increased *ERG* expression levels in the hematopoietic system of the mouse. Brady and colleagues reported that transplantation of fetal liver-derived murine hematopoietic stem and progenitor cells (HSPC) retrovirally overexpressing a human *ERG* ORF into sublethally irradiated mice resulted in fully penetrant megakaryoblastic leukemia.^54^ Tsuzuki and Seto^55^ found that transplantation of adult bone marrow (BM) cells from 5-FU-stimulated donor mice retrovirally expressing a human *ERG* ORF into lethally irradiated mice induced a leukemia-like disease characterized by accumulation of CD71^+^/Ter119^+^ erythroblasts and expansion of CD4^+^/CD8^+^ double positive T cells. Kile and colleagues reported that transplantation of fetal liver-derived or adult mouse bone marrow (BM) (from 5-FU treated donors) retrovirally expressing a murine *Erg* ORF into irradiated mice induced a leukemia-like disease. Similar to the observation by Seto, some mice developed CD4^+^/CD8^+^ T-cell leukemia, others developed nonlymphoid disease composed of CD71^+^/Ter119^+/−^ cells in some, but also CD71^+^/CD41^+^ cells in other mice.^85^ Collectively, these studies suggest that abnormally high *ERG* expression contributes to hematopoietic malignancies affecting the erythromegakaryoblastic and T-cell lineage.

The Sleeping Beauty (SB) transposon-based mutagenesis system allows to identify potentially cooperating genetic lesions for cancer development. Targeting a conditional SB allele to the hematopoietic system in mice expressing the constitutively active J*AK2*^*V617F*^ resulted in a strong phenotypic selection for an erythroleukemia-like disease.^86^ The vast majority of *SB/JAK2*^*V617F*^ mice developed an aggressive erythroleukemia occasionally coincident with CD4^+^/CD8^+^ T-cell ALL. Interestingly, the most prevalent common transposon insertion sites were the genes encoding for the transcription factors ERG and ETS1. Notably, transplantation of fetal liver-derived HSPC retrovirally expressing an AML-associated *TLS-ERG* fusion also induced a very similar erythroleukemia as observed in the *SB/JAK2*^*V617F*^ mice. Expression of *TLS-ERG* in SB mice resulted in acceleration of the disease. Interestingly, the *Jak2* gene locus was among the most common CIS in this model furthermore underlining cooperation of ERG and constitutively active JAK2 in murine erythroleukemia.^86^

##### Caudal-type homeobox 4

The caudal-type homeobox family comprises CDX1, CDX2, and CDX4 known as developmental regulators of the clustered HOX homeobox genes.^87^ In normal hematopoiesis, CDX4 mirrors *HOX* gene expression with a peak in hematopoietic stem cell and decreasing upon differentiation.^88^ Ectopic *Cdx4* expression in mouse embryonic stem (ES) increased the hematopoietic colony output associated with upregulation of a *Hox* gene expression.^89^ However, *Cdx4* gene inactivation only minimally affected adult hematopoiesis in mice.^90^ Retroviral *Cdx4* overexpression induced aberrant self-renewal potential in mouse hematopoietic cells in vitro and transplantation induced an AML-like disease in about 50% of mice.^91^

Feuring-Buske and colleagues recently reported *Cdx4* mRNA expression in a small cohort of AEL patients and in 3 established AML cell lines with an erythroid phenotype. Similar to previous studies, retroviral *Cdx4* overexpression provided aberrant serial replating potential to BM-derived hematopoietic cells.^57,91^ Notably, mice transplanted with *Cdx4* virally transduced BM-derived HSPC developed a transplantable erythroleukemia-like disease after a long latency, characterized by anemia, splenomegaly with infiltration of CD71^+^Ter119^+/−^ erythroid progenitors, occasionally erythroid progenitors in the periphery, and multiorgan infiltrations upon propagation into secondary recipients. Tumor cells were characterized by low expression levels of genes associated with erythroid specification or differentiation including *Gata1*. Interestingly, leukemic blasts from diseased mice carried some additional mutations in erythroid transcription factors like GATA1 or GATA2. These observations indicate that aberrant *Cdx4* expression levels in a permissive progenitor may induce a transcriptional program that interferes with normal erythroid development. However, the direct relevance for the human disease remains unclear, as the transcriptome analysis of large AEL patient cohorts did not highlight *Cdx4* alterations.^19,21^

##### EWS-FLI fusion

The *Fli-1* gene encoding for an ETS-transcription factor was identified as an additional common integration site in FLV-induced erythroleukemia.^92,93^ In addition to FLV-induced mouse erythroleukemia, *Fli-1* mRNA expression was found in some human AML cells lines with erythroid phenotypes.^94^ Experimental *Fli-1* overexpression was shown to reduce the expression of GATA1 and to impair induced erythroid differentiation in human and mouse cell lines.^95^ Apart from FLV-driven mouse erythroleukemia, *FLI-1* is better known as fusion partner to *EWSR1* (EWS) as consequence of a t(11;22)(q24;q12) chromosomal translocation found in Ewing’s sarcoma and other neuroectodermal tumors.^96^ Interestingly, *Mx1-iCre*-controlled hematopoietic expression of a transgenic *EWS-FLI-1* ORF in the *Rosa26* murine gene locus rapidly resulted in a highly penetrant aggressive transplantable erythroleukemia with tumor cells expressing Kit, CD71, CD43, and Gata1, but no Ter-119 or other lineage markers. Leukemic cells expressed high levels of *Myc* but did not harbor any gross chromosomal or *Trp53* alterations.^53^ Although the erythroleukemia phenotype does not match the human disease associated with this fusion, these transgenic mice provided an in vivo platform to study strategies for therapeutic targeting of *EWS-FLI1*-driven tumors.^97^ Notably, several compounds were found that inhibit Fli-1 transcriptional activities and impaired *EWS-FLI-1*-driven erythroleukemia in mice. Their detailed mode of action and clinical value for human erythroleukemia remains to be elucidated.^97,98^

#### Erythroleukemia by cooperating genetic lesions

##### BCR/ABL and loss of C/EBPα

Earlier work suggested that AML is the product of signaling mutations (eg, in tyrosine kinases) supporting proliferation and survival that functionally cooperate with mutations in hematopoietic transcription factor mutations blocking differentiation (Table 2).^104^ BCR-ABL is a constitutively active tyrosine kinase fusion associated with chronic myeloid leukemia (CML) and is also recurrently found in AEL.^10^ Tenen and colleagues developed a model of progression from chronic to acute disease by retrovirally expressing BCR-ABL in fetal liver-derived HSPCs lacking the myeloid transcription factor CCAAT-enhancer binding protein (C/EBPα).^99^ Mice transplanted with *BCR-ABL*-expressing *Cebpa*^−/−^ cells developed acute erythroleukemia with infiltration of BM and spleens and erythroblasts on peripheral blood smears. Furthermore, tumor cells expressed erythroid regulators such as SCL/TAL1 and GATA1. Notably, similar to tumor cells from diseased *BCR-ABL* transduced *Cebpa*^−/−^ mice, the human erythroleukemia cell line K562 (established from a patient with CML in blast crisis) also expresses BCR-ABL and GATA1 and lacks C/EBPα. Functional studies with *Cebpa*^−/−^ fetal liver progenitors revealed that C/EBPα functions in hematopoietic cell fate decisions by the dual actions of inhibiting erythroid and inducing myeloid gene expression.^105^

**Table 2.** Rational AEL Mouse Models Based on Genetic Cooperation

Year	Gene	Model	Phenotype	Major Findings	Surface Markers on Leukemic Cells	Reference
2010	*BCR-ABL*—*Cebpa*^−/−^	*MSCV* viral overexpression and BM reconstitution	Erythroleukemia after 26–157 d, 100% penetrance. Transplantable (5/6 mice)	Splenomegaly, erythroblasts on PB smears. Tumor cells expressed SCL/TAL1	CD71^+^, Ter119^−^	^99^
2013	*Sleeping beauty—JAK2*^*V617F*^	SB insertion tagging, *MSCV* viral overexpression and BM reconstitution	Erythroleukemia (75%), after a median latency of 50 d. Transplantable	Erythroblast infiltration in BM and spleen. ERG and ETS1 were the most frequent integration site	CD71^+^, Ter119^−/lo^, some CD41^+^ cells	^86^
2014	*JAK2*^*V617F*^—*Trp53*^−/−^	*MSCV* viral overexpression and BM reconstitution	Erythroleukemia -like, after 14–100 d. Transplantable	Hepatosplenomegaly, normal platelet counts	Kit^+^, CD71^+^, Ter119^+/−^	^23^
2017	*JAK2*^*V617F*^—*Trp53*^−/−^	*MSCV* viral overexpression and BM reconstitution	Erythroleukemia, 100% penetrance, median latency 46.5 d. Transplantable	Anemia, hepatosplenomegaly, pulmonary hemorrhage, and expansion of dysplastic erythroid progenitors	CD71^+^, Ter119^−^	^100^
2019	*NTRK1*^*H498R*^—*Trp53*^*R173H*^	*MSCV* viral overexpression and BM reconstitution	Erythroleukemia, 100% penetrance, median latency 25 d. Transplantable	Hepatoplenomegaly, infiltration of GATA1^+^, RUNX1^+^, Ter119^+/−^ tumor cells. Tumor cells were sensitive to Lacrotrectinib	CD71^+^, Ter119^+/−^	^19^
2019	*Gata2—Cebpa*^*K/L*^	*Cebpa*^*K/L*^; *Gata2*^*G320D/+*^ knock-in alleles, fetal liver hematopoietic cell transplants	Bi-lineage acute erythroleukemia almost 100% penetrance, after 8–10 mo	Anemia, thrombocytopenia, and splenomegaly	Kit^+^, CD71^high^, Ter119^low^, but also some Mac1^+^ cells	^101^
2020	*Gata1s—Tet2*^−/−^	Erythroblasts from *Tet2*^−/−^ *+ Gata1s* transgenic mice grown in vitro and transplanted	Erythroleukemia, 100% penetrance	Anemia, Hepato-splenomegaly, Infiltration of erythroblast GATA1^+^ in BM, spleen, and liver	CD71^high^, Ter119^+/−^	^21^
2020	*ERG—TP53*^*R248Q*^	*MSCV* viral overexpression and BM reconstitution (HUPKI). EB sorting from the first degree recipients and injected into the second degree recipients	Erythroleukemia, 100% penetrance with median latency 60 d (2^nd^ recipients)	Anemia, Hepatosplenomegaly, Infiltration of erythroblast GATA1^+^ in BM, spleen, and liver	CD71^high^, Ter119^+/−^	^21^
2020	*NFIA-ETO2*—*TP53*^*R248Q*^	*MSCV* viral overexpression and BM reconstitution (HUPKI)	Pure erythroid leukemia 80%–100% penetrance. Fully transplantable	Anemia, hepatosplenomegaly, organ infiltration, erythroblasts on blood smears	Kit^+^, CD71^+^, Ter119^+/−^	^102^
2021	*Tp53/Bcor/Dnmt3a and Tp53/Bcor/Rb1/Nfix*	Lentiviral vectors with different of sgRNA for multiplex genome editing in Cas9-eGFP- lineage-negative HSCs and BM reconstitution	Erythroleukemia	AEL cells sensitive to CDK9 inhibitor (LY2857785)	Not yet access	^103^

AEL = acute erythroleukemia; BM = bone marrow; MSCV = murine stem cell virus.

##### GATA2 and C/EBPα mutations

Genetic alterations of the C/EBPα and GATA2 transcription factors regulating myeloid differentiation and HSC self-renewal have been reported in some AEL patients.^17,19^ A recent mouse model demonstrated that bi-allelic *Cebpa* mutations led to myeloid leukemia development and that an additional *Gata2* mutation enhanced leukemogenesis with a subset of triple transgenic mice (40%) developing leukemia with erythroid and myeloid features.^101^ Interestingly, the identified leukemia-initiating cells in both models were neutrophil-monocyte progenitors and molecular characterization of this population revealed distinct function of each cooperating mutations. While bi-allelic *Cepba* mutations increased expression of erythroid genes, the *Gata2* mutation increased chromatin accessibility at erythroid TF motifs (eg, GATA1, ZFPM1, and KLF1) and decreased it at myeloid TF motifs. These findings suggested that the erythroid phenotype of this leukemia model is driven by the aberrant chromatin accessibility at key erythroid TF-regulated loci, controlled by aberrant GATA2 activity.

##### JAK2^V617F^ and loss of Trp53

The majority of BCR-ABL-negative MPNs are driven by somatic activating mutations of the JAK2 tyrosine kinase of which V617F is the most prevalent. A subset of MPN patients progress to AML that is associated with recurrent somatic alterations affecting epigenetic regulators, splicing-related factors and/or the TP53 tumor suppressor.^106^ To demonstrate potential cooperation, researchers retrovirally overexpressed *JAK2*^*V617F*^ in either wildtype or *Trp53*^−/−^ BM-derived HSPCs and transplanted them into irradiated wildtype recipients.^23,100^ Mice developed a serially transplantable leukemic phenotype with hepatosplenomegaly with infiltration of CD71^+^/Ter119^−^ erythroid progenitor cells. Kurokawa and colleagues found abnormal karyotypes, such as hyperdiploidy, suggesting increased genomic instability upon *Trp53* loss.^100^ Viral expression of wildtype *Trp53* significantly reduced clonogenic activity of in vitro and in vivo leukemia induction by *Jak2*^*V617F*^*;Trp53*^−/−^ erythroblasts suggesting an active role in aberrant self-renewal. Both groups explored therapeutic approaches in their models. Kurokawa and colleagues reported that a potent JAK1/2 inhibitor (INCB18424) reduced the spleen weight but did not affect tumor cells in the BM.^100^ Levine and colleagues found that treatment with the ruxolitinib JAK2 inhibitor somehow prolonged the survival of secondary recipients of *Jak2*^*V617F*^*;Trp53*^−/−^ erythroblasts; however, treatment with an HSP90 inhibitor appeared more efficient in reducing tumor cell load and restoring normal myelopoiesis.^23^ Although these studies demonstrated cooperation of *Jak2*^*V617F*^ and loss of *Trp53* in mice, post-MPN erythroleukemia patients mostly develop *TP53* DNA-binding domain point mutations rather than loss of both alleles that seemed to be essential for the observed mouse phenotype in these experiments.

##### NTRK1 and mutated TP53

*NTRK1* is a member of the neurotrophic receptor tyrosine kinase gene family including the TRK-A, TRK-B, and TRK-C receptors, respectively. NTRKs are target of gene fusions, deletions/truncations and point mutations not only in hematological malignancies but also in various solid cancers that mostly lead to constitutive activity.^107,108^ Iacobucci et al^19^ found NTRK1 tyrosine kinase domain (H498R, G6117D, and H766R) mutations in tumor cells from 3 AEL patients carrying *TP53* mutations. While activation of downstream signaling pathways is likely dependent on both the cell context and the type of NTRK mutation, NTRK fusion expression in hematopoietic progenitors led to increased phosphorylation of AKT and PLCγ1.^108^ Together with the observation that PI3K-AKT mutations have been found in about 7% of AEL (primarily older adults), aberrant activation of this pathway may be a driver of some human AEL.^19^ NTRK1 mutations in AEL gained particular attraction as selective small molecule TRK inhibitors (TRKi) such as larotrectinib, which has been shown to have clinical activity in cancer patients.^109^ To model functional cooperation, Iacobucci and colleagues transplanted wildtype and *Trp53*^*R172H*^ BM-derived HSPCs retrovirally expressing wildtype or mutant *NTRK1* into irradiated syngeneic recipient mice. *Trp53*^*R172H*^ is the murine homolog to human *TP53*^*R175H*^ one of the most frequent cancer-associated TP53 DNA-binding domain mutations. Notably, expression of wildtype or mutant *NTRK1* in *Trp53*^*R172H*^ cells resulted in a transplantable erythroleukemia-like disease. Expression of the *NTRK1*^*H498R*^ did result in significantly earlier disease than overexpression of wildtype *NTRK1*. Notably, *NTRK1/Trp53* comutant tumor cells appeared very sensitive to in vivo TRKi therapy. Whereas vehicle-treated mice rapidly succumbed to the disease, larotrectinib-treated mice did not develop any disease >100 days posttransplant. Although NTRK mutations are relatively rare, these observations suggest that, like other cancers carrying NTRK alterations, treatment with selective TRK inhibitors could be of therapeutic benefit for AEL patients carrying these alterations.

##### NFIA-ETO2 and mutated TP53

The t(1;16)(p31;q24) chromosomal translocation found in PEL from very young children leads to fusion of the nuclear factor 1A (NFIA) to ETO2 (also known as CBFA2/RUNX1 Partner Transcriptional Co-Repressor 3, CBFA2T3).^29–33^ The transcription factor NFIA has previously been shown to control erythroid fate of hematopoietic progenitors, while ETO2 is as transcriptional cofactor controlling HSC and differentiation of erythroid progenitor cells.^110,111^ We observed that retroviral overexpression of *NFIA-ETO2* fusion blocked in vitro erythroid differentiation of MEL cells and primary murine erythroblasts. However, *NFIA-ETO2*-expressing cells could not be serially propagated in growth-factor containing methylcellulose and transplantation of *NFIA-ETO2*-expressing BM or fetal liver erythroid progenitor cells into irradiated mice did not result in any disease. In contrast, *NFIA-ETO2*-expressing erythroblasts harboring one of the most frequent cancer and AEL-associated TP53 mutation, *TP53*^*R248Q*^, could be serially plated in MC and when transplanted into irradiated recipients, induced a fully penetrant transplantable lethal erythroleukemia-like disease characterized by hepatosplenomegaly, anemia, thrombocytopenia, and the presence of erythroid progenitor cells on peripheral blood smear.^102^ Molecular studies suggested that NFIA-ETO2 primarily blocks erythroid differentiation by repressing NFIA as well as GATA1 target genes, and that the TP53^R248Q^ mutation endowed cells with aberrant stemness and aberrant activity of the polycomb complex 2 (PRC2). In addition, similar to other ETO-protein containing fusions, *NFIA-ETO2* immortalized cells may also be sensitive to small peptides that disrupt ETO-NHR-domain-mediated protein/protein interactions, suggesting a potential therapeutic vulnerability.^102,112^

##### Alterations of BCOR collaborating with TP53 and DNMT3A mutations

To functionally demonstrate oncogenic cooperation, Iacobucci and colleagues used multiplexed CRISPR/Cas9-mediated genome editing of HSPCs followed by BM reconstitution in irradiated mice.^103^ They established 14 genetically different leukemia mouse models in which induction or an AEL phenotype was associated with inactivation of the *Bcl-6 co-repressor* (*Bcor*) and *Trp53* either alone or co-mutated with *Dnmt3A*, *Retinoblastoma 1 (Rb1*) or *Nuclear factor I X (Nfix1*). Erythroleukemia in the mice was characterized by aberrant expression of erythroid developmental regulators such as *Gata1*, *Kruppel-like factor 1* (*Klf1*), or *Nuclear factor erythroid*-2 (*Nfe2*), driven by the interaction of mutations of the epigenetic modifiers *Dnmt3a* and *Tet2* that perturbed methylation and thus expression of lineage-specific transcription factors. Putative loss of function BCOR mutations represent a substantial fraction of cytogenetically normal (CN)-AML patients, are frequently associated with DNMT3A mutations and were proposed to be associated with an inferior outcome.^113^ Sportoletti et al^114^ explored functional cooperation with a conditional mouse model mimicking AML-associated BCOR truncating mutations. They observed expansion of erythromegakaryocytic progenitors, anemia, and thrombocytosis but no overt leukemia in *Bcor*^−/−^ mice. In contrast, all *Bcor/Dnmt3A* double knockout mice developed AEL-like leukemia characterized by the expansion of Kit^+^/Ter119^+^ cells. Interestingly, the gene expression signatures of the leukemic cells suggested functional interference with GATA1-regulated erythroid differentiation.^114^ Both of these studies demonstrated functional cooperation of *Bcor* alterations with *Trp53* and/or *Dnmt3A* in mice; however, BCOR mutations seemed to be very rare events in human AEL.^14–21^ Interestingly, murine *Bcor/Trp53*-mutated AEL cells were sensitive to the PARP inhibitor talazoparib and the demethylating agent decitabine, and combined *Trp53/Bcor/Dnmt3a* mutation conferred sensitivity of AEL cells to CDK7/9 inhibitors.^103^

#### Unexpected erythroleukemia phenotypes in genetically modified mice

##### Inactivation of the HELLS/lymphoid specific helicase chromatin remodeler

HELLS (also known as lymphoid specific helicase or SMARCA6) is a member of the SNF2 subfamily of helicases mostly known for their chromatin remodeling activity. It is thought that the HELLS protein controls the access of de novo methyltransferases DNMT3A/B mostly at DNA repeat elements but also at selected targets including the *Hox* gene loci (Table 3).^118^ HELLS also seems to modulates chromatin binding of lineage-specific transcription factors including GATA3, SCL/TAL1 or E2A.^115^ *Hells*^−/−^ mice have multiple defects (growth retardation, premature aging phenotype) and mostly die prematurely.^119^ To address the role of HELLS for normal hematopoiesis, Muegge and colleagues transplanted fetal liver-derived cells from *Hells*^−/−^ mice into irradiated recipients. Interestingly, recipient mice developed hematologic malignancies including lymphoma or an erythroleukemia-like disease. Although only about 10% of the mice developed this disease, over 50% of them had signs of abnormal erythropoiesis. Although no detailed characterization was reported, the tumor cells appeared to express erythroid CD71 and Ter119 markers. Molecular analysis revealed global DNA hypomethylation and de-repression of endogenous retroviral repeats. In addition, increased expression of *Spi1* mRNA and protein was found in *Hells*^−/−^ fetal livers. The loss of *Hells* was associated with reduced Dnmt3b binding to retroviral elements within the PU.1 gene suggesting that HELLS generally controls Dnmt3B binding to chromatin.^120^ The human HELLS homolog (also known as proliferation-associated SNF2-like gene product) is widely expressed in AML and ALL cell lines and primary samples. Interestingly, a 25-bp mRNA deletion lacking a region critical for transcriptional activation of the yeast homolog was detected in about half of the leukemia samples tested but not in other cancers; however, its functional significance remains unclear.^121^

**Table 3.** Unexpected AEL mouse model

Year	Gene	Model	Phenotype	Major Findings	Surface markers on leukemic cells	Reference
2008	*Hells*^−/−^	Constitutive gene knockout	*Hell*^−/−^ fetal liver hematopoietic cell transplant. 7% erythroleukemia, some lymphoma. 11% erythroleukemia upon co-deletion of *Trp53*	Anemia, splenomegaly, infiltration by erythroblasts	Relative increase in CD71^+^/Ter119^+^ cells	^115^
2019	*iH3*^*K36M*^	Dox-inducible (*rtTA, Rosa26*), transgene in *Col1A1* locus	Lethal hematologic disorder after median latency of 50 d	Anemia, thrombocytopenia, splenomegaly, accumulation of erythroid progenitors. Relative BM hypocellularity	Increase in colony forming unit-erythroid progenitors and CD71^+^ proerythroblasts, expressing low levels of c-Kit. Decrease in Ter119^+^ EBs	^116^
2020	*Nsd1*^−/−^	Targeted gene knockout (*Vav-iCre; Nsd1*^*fl/fl*^)	Pure erythroleukemia-like disease, transplantable, 100% penetrance	Anemia, thrombocytopenia, splenomegaly, erythroblasts on peripheral smears. Multiorgan infiltration and relative BM hypocellularity	CD71^low^, Kit^+/−^, FcγRII/III^+/−^, CD34^−^, B220^−^ and Sca-1^−^	^117^

AEL = acute erythroleukemia; BM = bone marrow.

##### Inactivation of the nuclear receptor interacting SET domain 1 methyltransferase

Posttranslational modification of the histone tails is one of the key events of epigenetic gene regulation. Hereby, trimethylation of lysine 4 and 36 of histone 3 are generally associated with active transcribed regions, whereas trimethylation of lysine 9 or 27 correlates with repression of a given gene locus. The so-called epigenetic code is based on the interplay of histone lysine methyltransferases (HMT) that set these marks (referred as “writers”) and demethylases (HDM) that remove them (referred as “erasers”). Multiple genes encoding for these epigenetic regulators are targets of recurrent somatic gene alterations that have been shown to contribute to AML initiation and maintenance.^122^ H3K36me3 is the product of mono- and dimethylation by the nuclear receptor interacting SET domain (NSD)1–3 family, ASH1L, or SETMAR followed by SETD2-mediated trimethylation.^123^ NSD1 is the target of recurrent genomic alterations in human cancers. Highly prevalent putative loss of function *NSD1* mutations have been found in head and neck and other solid cancers, and *NSD1* expression was reported epigenetically silenced in renal carcinomas.^124^ In contrast to solid cancers, NSD1 mutations are rare in hematological malignancies; however, *NSD1* was found to be target of a recurrent t(5;11) chromosomal translocation found in childhood AML that results in a fusion with the *NUP98* gene.^125^ To better understand its function in hematopoiesis, we inactivated *NSD1* in human and mouse hematopoietic cells.^117^ Hematopoietic (*Vav1-iCre*-mediated) inactivation of *Nsd1* induced a fully penetrant erythroleukemia-like disease characterized by anemia, thrombocytopenia, splenomegaly, and multiorgan infiltrations with occasional erythroblasts on peripheral blood smears. *Nsd1*^−/−^ erythroblasts formed abnormal serially replating burst forming unit-erythroid in EPO-containing MC. Transplantation of BM cells from diseased mice propagated the disease in wild-type recipients, alone or in competition with normal cells. Despite constitutive expression of the erythroid master regulator GATA1, in vitro erythroid terminal maturation of *Nsd1*^−/−^ erythroblasts was significantly impaired. Expression of known positively regulated GATA1 targets was decreased, while the regulation of GATA1-repressed target genes was less affected. Retroviral overexpression of *Gata1* was able to overcome the terminal differentiation block in vitro. Similarly, retroviral expression of wildtype, but not a catalytically inactive *Nsd1*^*N1918Q*^ mutant, was also able to rescue the terminal maturation block associated with upregulation of erythroid differentiation-associated genes on the mRNA and global proteome level. Despite very similar *Gata1* mRNA and protein levels, only *Nsd1*^−/−^ erythroblasts expressing wildtype *Nsd1* showed significantly increased binding of Gata1 to many known target genes. These observations suggested that the catalytic activity of NSD1 is an essential permissive factor for proper transactivation of GATA1 targets for productive terminal erythroid maturation. Importantly, knockdown of *NSD1* mRNA significantly altered the clonogenic growth of human CD34^+^ HSCP leading to accumulation of immature erythroid progenitor cells strongly suggesting that independent of the species, NSD1 activity controls terminal erythroid maturation.

##### Hematopoietic expression of an inducible H3^K36M^ oncohistone transgene

H3K36 is target of multiple aberrations in human cancer including mutations miswriting the marks or aberrant expression of the respective HMT, but also by mutations of the.^126^ Originally identified in the histone 3.3. (H3.3.) variant in chondroblastoma, H3 lysine (K) to methionine (M) mutations were later also found in H3.1 in several human cancers (also known as oncohistones) including pediatric soft-tissue sarcomas and some head and neck squamous cell carcinoma. The H3^K36M^ mutant protein seems to sequester the SETD2 HMT resulting in globally reduced H3K36me3 marks but also inhibits other HMTs active on H3K36 such as NSD1 and NSD2 resulting in reduced H3K36me1/2.^127^ To address the impact of a H3^K36M^ mutation in primary cells in vivo, Hochedlinger and colleagues established ES cells and mice with Doxycycline (DOX)-induced overexpression of a *H3*^*K36M*^ transgene integrated in the *Col1A1* gene locus.^116^ Adult *iH3*^*K36M*^ transgenic mice developed symptoms of disease after 4–7 weeks on DOX. In addition to nonhematopoietic aberrations (testicular atrophy, lack of Paneth cells in the intestine), the mice developed thymic atrophy, splenomegaly, and reduced BM cellularity. They presented with anemia, thrombocytopenia, and increased white blood counts with erythroid progenitors in the periphery mimicking acute erythroleukemia. Gene expression profiling revealed an increased expression of regulators of the erythroid lineage and downregulation of genes known to control HSC and/or myelopoiesis. Notably, the H3K36me3 mark was depleted in downregulated genes of which some showed increased H3K27me3 marks at promoter and adjacent to gene bodies. Overall, there was a correlation between loss of H3K36me and decreased chromatin accessibility, but only modest decreases in DNA methylation were observed over gene bodies of some hematopoietic regulators. The erythroleukemia phenotype was strikingly similar between *iH3*^*K36M*^ and *Nsd1*^−/−^ mice, which underlines that appropriately regulated H3K36 methylation is critical for terminal erythroid differentiation.

## Emerging molecular mechanism of erythroleukemia

### Is GATA1 a core player in the pathogenesis of human erythroleukemia?

GATA1 is a master regulator of normal erythropoiesis acting in transcriptionally active complexes with TAL1, LMO2, LDB1, RUNX1, ETO-, and ETS-family proteins.^67,128^ GATA1 undergoes multiple posttranslational modifications including phosphorylation, acetylation, sumoylation, and ubiquitination that in part all control its transcriptional activity.^129^ Mutational and transcriptomic analysis of primary human AEL cells supports the idea that several alterations may converge on the functional alteration of GATA1 through various mechanisms. First, some transcriptionally active proteins of the GATA1 complexes, including GATA1 itself, are targeted by mutations or part of fusion genes in AEL (eg, GATA1s, NFIA-ETO2, or MYB-GATA1).^19,21,29–33^ However, these alterations are rare and therefore do not account for the erythroid phenotype of the majority of AEL. Second, mouse models have shown that altering the expression of several factors, including ectopic expression of ERG, SPI1, or FLI1 or reduced expression of GATA1 can induce AEL phenotypes, indicating that additional mechanisms converging on GATA1 lead to the development of erythroid leukemia.^50,52,85,93^ In the line of these observations in mice, we found that aberrantly high expression of several proteins that impact GATA1 function are recurrent alterations in human primary AEL cells. This includes high expression of factors like ERG, CBFA2T3, or SKI that functionally antagonize GATA1-dependent differentiation in G1E cells. Notably, ectopic expression of these transcription factors in mouse erythroblasts resulted in their immortalization associated with decreased chromatin accessibility at GATA1 binding sites.^21^

In the 2 AEL cohorts for which transcriptome data are available, the subset of AEL samples presenting alterations of expression of either transcription factors or signaling intermediates impacting GATA1 activity represents up to 25% of cases.^21^ Notably, aberrantly high expression levels of ERG or EPOR were also reported in another independent AEL cohort resulting from *bona fide* genomic amplifications including ERG and EPOR genes.^21,130,131^ Overall, these findings strongly support a functional convergence on aberrant GATA1 activity in human AEL either through direct genetic alterations (eg, in regulatory regions to be identified) or as a result of an epigenetic drift as outlined in the following section.

Mechanistically, aberrant GATA1 activity may result in at least 2 cellular consequences. First, inhibition of GATA1 activity may derive from distinct mechanisms involving aberrant maintenance of GATA1 transcriptional repressor (eg, ETS-associated or ETO2 transcriptional complexes), GATA1 destabilization at the protein levels through alterations of GATA1 posttranslational modifications or aberrant protein-protein interaction, or by alterations affecting GATA1 chromatin binding. These mechanisms would contribute to prevent erythroid progenitor differentiation progression toward fully mature erythroid cells. Second, GATA1 activity could be aberrantly activated or maintained, including through constitutive activation of signaling factors (eg, through JAK2^V617F^ or high EPOR expression), leading to the abnormal commitment of early immature progenitors toward the erythroid lineage. This idea is supported by the recent model combining bi-allelic *Cebpa* and *Gata2* mutations.^101^ Hereby, *Cebpa* and *Gata2* mutations synergize by increasing erythroid transcription factor (*Gata1, Klf1,* and *Zfmp1*) expression and erythroid chromatin access, respectively, thereby installing ectopic erythroid potential. In addition, while bi-allelic *Cebpa* mutation resulted in increased erythroid transcription factors expression, it also led to increased expression of several erythroid repressors including *Gata2*, *Erg*, or *Cbfa2t3*. These findings collectively suggest that a combination of these 2 antagonistic mechanisms on GATA1 activity in the same progenitor could explain both, the erythroid bias and the blockage of differentiation. The degree of erythroid commitment may depend on both the place of the targeted progenitor in the hematopoietic hierarchy and the cooperating mutations. Of note, GATA1 is also mutated in myeloid leukemia of Down syndrome (ML-DS) that frequently present with erythroid cell marker expression, and is generally considered at the frontier between megakaryoblastic and erythroid leukemia.^132^ While numerous studies have addressed GATA1 function, it is likely that further analyses are required to fully understand how the activity of GATA1 is fine-tuned and how these alterations contribute to erythroid transformation.

### Impaired TP53 activity and malignant erythropoiesis

TP53 regulates HSPC quiescence and self-renewal; thus, impaired function of TP53 promotes HSPC proliferation that likely leads to additional DNA damage and hematopoietic malignancies.^133,134^ Although *TP53* mutations represent by far the most frequent mutated genes in AEL and particularly in PEL, no model clearly demonstrated yet a link with the erythroid phenotype. Impaired erythropoiesis in MDS carrying a deletion on 5q has been linked to activation of TP53 upon inactivation of the ribosomal protein small subunits (RPS)-14 or -19.^135^ More recently, activation of TP53 during ribosomal biogenesis have been proposed to regulated normal erythroid differentiation.^136^ Most *TP53* alterations identified in AEL and other AML subtypes are missense mutations in the DNA binding domain but their functional consequences (ie, inactivating, gain-of-function or dominant negative) remain a matter of debate. Functional studies in mice suggested that *TP53* mutations are drivers of clonal hematopoiesis.^137^ Several TP53 DNA-binding domain mutations have been reported to disrupt the structure and activity of the protein, allowing neomorphic interactions with several tumor suppressive factors, including TP73 that abrogate its function.^138^ In addition, such potentially TP53-gain-of-function mutants appear to be stabilized by binding to HSP90 leading to the inactivation of MDM2 and CHIP E3 ligase-mediated degradation.^139^ Of importance, these molecular mechanisms could be pharmacologically blocked by small-molecules interfering with TP73 and HSP90.^134,140^ However, more recent functional studies suggested that the most prevalent AML-associated TP53 mutations act in a dominant-negative manner rather than gain-of-function.^141^

TP53 interferes with the activity of multiple transcription factors. For example, some TP53 mutants have been shown to interact with the ETS1 transcription factor leading to increased expression of multidrug-resistance 1 (MDR1) associated with a poor outcome in AML.^142^ Experimental evidence linked TP53 to GATA1 activity in normal erythropoiesis. TP53 and GATA1 may interact, through their transactivation domain and DNA-binding domain, respectively, leading to mutual inhibition of their transcriptional activities.^143^

TP53-DNA-binding domain mutations were shown to interact with epigenetic factors, including EZH2, KMT2A, KMT2D, and KAT6A. Notably, TP53 mutants bind to and enhance EZH2 chromatin association, resulting in an increased level of H3K27me3 at essential regulator of HSC function and differentiation.^138^ We recently found that the PEL-associated *NFIA-ETO2* fusion gene functionally cooperated with one of the most prevalent AEL-associated *TP53*^*R248Q*^ mutation most likely also through functional interference with the PRC2 complex.^112^ TP53-DNA-binding domain mutations may also interact and enhance the activity of the methyltransferase KMT2A/2D and the acetyl transferase KAT6A leading to increase genome-wide methylation and acetylation.^144^ Consistently, KMT2A/2D and KAT6A are upregulated in mutant TP53 AML and are often mutated in AML, including AEL.^19,145^ While additional work is necessary to dissect the link between TP53 mutation and epigenetic gene regulation, it could open the avenue for novel therapeutic avenues. If TP53 mutants indeed enhance KMT2A activity through KMT2A partners such as MEN1, TP53-mutant leukemia including AEL may benefit from the recent development of highly potent and selective small molecules blocking the functionally critical interaction of KMT2A and Menin.^146^

In MDS, TP53 mutations are generally associated with high-risk disease, rapid transformation to AML, therapy resistance, and poor outcome. Studying a large MDS patient cohort, Papemmanuil and colleagues recently found that two-thirds of the patients had multiple hits indicating biallelic targeting which was predictive for leukemic transformation and early death. Interestingly, monoallelic patients die not differ from TP53 wildtype patients in outcomes and therapy response which would not really support a dominant-negative activity of these mutations (at least in the context of MDS).^147^ However, in at least 80% of TP53-mutated AML patients, more than 1 genetic alteration is present, reflecting the requirement for different oncogenic cooperation mechanisms.^135^ Indeed, TP53 mutations were shown to cooperate with multiple cellular signaling pathways. Loss of TP53 activity has been shown to cooperate with the KRAS^G12D^ activating mutation, inducing an aggressive AML in mice and with NRAS^G12D^ to promote megakaryocytic-erythroid progenitor (MEP) transformation leading to AML.^148,149^ The RAS signaling pathway is target of recurrent mutations in AEL.^19,21^ Similarly, as outlined before, mouse models have shown that genetic TP53 inactivation cooperates with several mutations, such as loss of function alterations of CEBPA or BCOR or constitutively active mutated tyrosine kinases such as JAK2^V617F^ or NTRK1^H498R^ to induce erythroleukemia in mice.^19,23,100–103,114^ Hence, treatment of xenografted AEL patient cells carrying either JAK2 or EPOR amplification with a JAK2 inhibitor significantly suppressed cell growth and prolonged overall survival.^130^ Together, these observations strongly suggest that TP53 mutations are essential players not only in AEL development but also crucial for the maintenance of the disease. However, it remains to be elucidated how TP53 mutations functionally interfere with transcriptional control of erythroid differentiation.

### Aberrant chromatin organization and erythroleukemia

AEL mouse models as well as sequencing of human samples strongly suggest that several proteins that control chromatin architecture by DNA methylation or histone modification play a central role in erythroid malignancies. DNA methylation is regulated by several factors including TET2, DNMT3A/B, or HELLS/Lsh.^150^ While DNMT3A/B is involved in de novo methylation by transferring a methyl group S-adenosyl-L-methionine to carbon position 5 of the nucleotide cytosine (5-mC), TET2 controls demethylation through the oxidation of 5-mC in 5-hmC. Mutational inactivation of these factors is recurrently observed in patients with MDS and AML resulting in enhanced HSC self-renewal and a decline in the output of differentiated progeny, thus predisposing to leukemic transformation in mice.^151^ Inactivation of *Tet2* or *Dnmt3a* in HSC resulted in increased myeloid and decreased erythroid gene expression signature resulting in aberrant accumulation of erythroid progenitors in mice. Notably, DNMT3A and TET2 were suggested to regulate hematopoietic differentiation by controlling accessible binding sites for distinct transcription factors including GATA1.^152–158^ In addition, very recent work has shown that precise DNA methylation patterning can control binding and regulation of GATA1 activity.^159^ Interestingly, a novel signaling pathway has been characterized that links TET2 activation to JAK2-mediated phosphorylation resulting not only in increased cytosine hydroxy-methylation and genome-wide loss of cytosine methylation but also enhanced interaction with the erythroid transcription factor KLF1.^160^ Interestingly, combined TET2 and DNMT3A inactivation, as frequently found in AEL, was also reported to increase of KLF1 and EPOR expression.^161^ As outlined above, genetic inactivation of *Dnmt3A* in mice lacking the transcriptional coregulator *Bcor* shifted the phenotype from macrocytic anemia to an erythroleukemia-like disease.^114^ Based on these observations, we speculate that the erythroid phenotype in some AEL cases is based on aberrant DNA methylation that impairs the erythroid transcriptional program. Aberrant DNA methylation is therapeutically targeted by cytidine analogs (eg, 5-Azacytidine or Decitabine) that incorporate DNA instead of deoxycytidine, covalently bind the enzyme and lead to DNMT degradation. Although a rather low specificity, positive clinical effects resulted into FDA approval to treat MDS and AML patients.^162^ Notably, earlier case reports of positive therapeutic responses to azacytidine were supported in a larger retrospective study including over 80 AEL patients treated with hypomethylating agents.^163^ Although a deeper molecular understanding of these effects would be important, it appears that similar to other AML forms HMAs as single agents are not curative for AEL.

Unexpected erythroleukemia models emerged from the inactivation or mutations of several chromatin-associated factors. Inactivation of the histone H3K36me1/2 methyl-transferase NSD1 leads to uncontrolled accumulation of erythroid progenitor cells resulting in a fully penetrant erythroleukemia-like disease in mice. Molecular analyses revealed a decrease of GATA1 target genes expression without significant expression changes in known GATA1-repressors suggesting that NSD1 is an essential epigenetic modulator of GATA1 target genes.^117^ Of importance, NSD1 has been identified being mutated in some cases of pediatric AEL either through an NUP98-NSD1 fusion or a NSD1 loss-of-function mutation found in a single patient.^19^ Notably, the *NSD1* gene is located on the long arm of chromosome 5, a region that is most frequently target of cytogenetic alteration in human erythroleukemia.^5,10^ As outlined above, inducible hematopoietic overexpression of an *H3*^*K36M*^ transgene resulting in reduced H3K36me1/2 methylation induced a very similar erythroid phenotype in mice as observed upon NSD1 inactivation. Molecular characterization of *H3*^*K36M*^ overexpressing HSPC revealed an expression of an aberrant erythroid signature, but the putative relation to GATA1 target activation remains unknown.^116^ Extensive biochemical in vitro experiments revealed that NSD1-mediated H3K36me2 marks are required for the recruitment of DNMT3A and maintenance of DNA methylation.^164^ Genetic ablation of *Nsd1* and its paralog *Nsd2* in murine cells resulted in a redistribution of DNMT3A to H3K36me3-modified gene bodies and a reduction in the methylation of intergenic regions. Notably, both, blood samples from individual with SOTOS overgrowth syndrome (carrying germline *NSD1* loss of function mutations) as well as NSD1-mutant cancer cells exhibited hypomethylation of intergenic DNA. This suggests that reduced H3K36 methylation connects human cancers and developmental overgrowth through aberrant intergenic CpG methylation.

Another chromatin modifier that has also been recently found to interact with GATA1 and other components of the GATA1-transcriptional complex is the KDM5A histone H3K4me demethylase.^165^ Notably, NUP98-KDM5A fusions have been found in pediatric AMKL and AEL patients.^19,35^ Although the ectopic expression of this fusion in human cord blood HSPC lead to immortalization and multilineage leukemia when injected in mice its impact on the chromatin and on GATA1 activity remains unknown.^166^

Chromatin organization is also orchestrated by the cohesin protein family, frequently mutated in myeloid malignancies including AEL.^19,21,130,167^ Mutations in cohesin-encoding genes are strongly associated with GATA1 mutations in ML-DS.^168,169^ Overall, cohesin mutations are associated with a global decrease in chromatin accessibility, but a relative enhancement of chromatin accessibility at binding sequences for some master hematopoietic stem-cell transcription factors such as GATA2, RUNX1, and ERG involved in myeloid transformation.^170^ Importantly, cohesin deficiency severely impaired erythroid differentiation of a multipotent cell line and enhanced self-renewal programs.^171^ These observations suggest that altered cohesion function may support erythroid transformation; however, their role for induction or maintenance of AEL remains to be elucidated. Collectively, these data support the idea that erythroid differentiation is tightly linked to chromatin organization and that aberrant expression or mutations of the regulatory proteins may lead to erythroid malignancies. While relatively few direct erythroid regulatory genes have been identified mutated in AEL, aberrant activity of epigenomic factors that control erythroid differentiation on the chromatin level are therefore valuable candidates to explain the erythroid phenotype of the disease.

**Figure 3.**
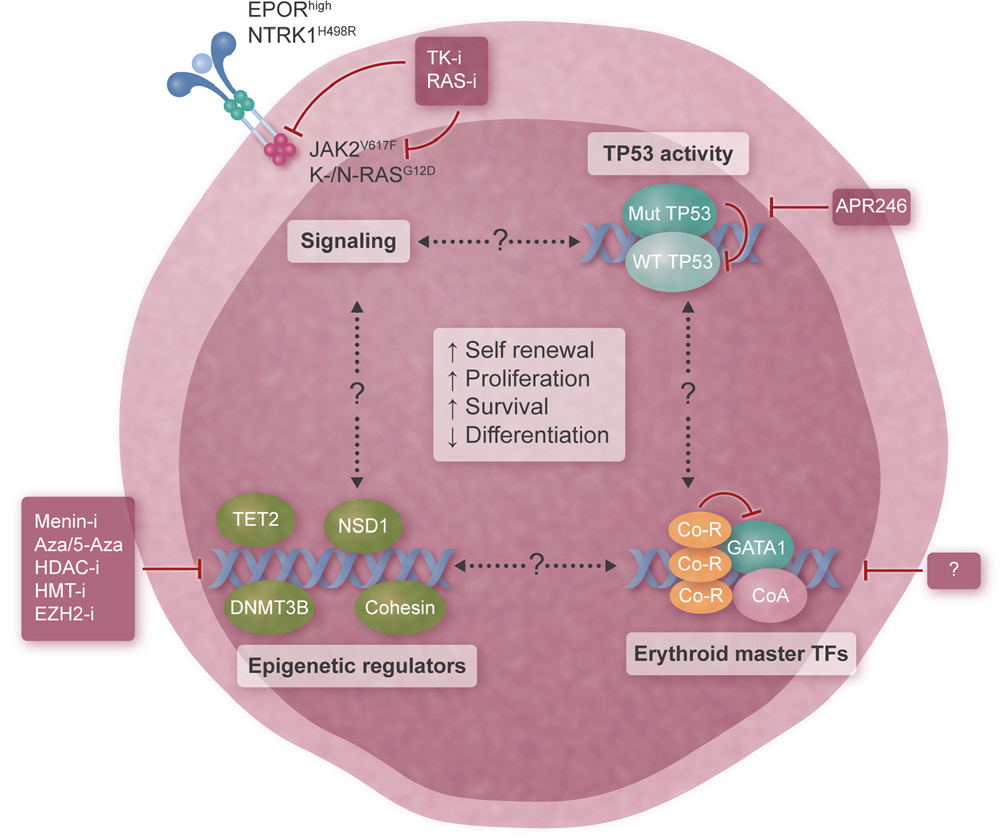
**Targeted therapeutic strategies emerging from the epigenomic landscapes and AEL disease models.** Schematic representation of major players identified to date in human AEL and the potential strategies for targeted interference, including (1) blocking aberrant activation of the JAK/STAT signaling axis (upper left); (2) restoration of the tumor suppressive TP53 activity (upper right); (3) inhibition of epigenetic regulators (lower left); and (4) reactivation of impaired activity of erythroid master transcription factors (like GATA1) to boost terminal differentiation of malignant erythroblasts (lower right). AEL = acute erythroleukemia.

Taken together, insights from multiple mouse models as well as the epigenomic landscape recently defined by deep sequencing and transcriptomics studies suggest that AEL reflects a disease continuum between MDS and AML that is characterized by a unique erythroid identity. While the molecular links between genetic/transcriptomic/epigenetic AEL alterations network remains to be elucidated (Figure 3), several therapeutic opportunities can be envisioned. Interference with the most prevalent genetic lesions like TP53 DNA binding mutations but also inhibition of classical signaling pathways, including the JAK/STAT pathway supports growth and survival, appears of primary interest and will require further preclinical assessments. In addition to these signaling nodes that are drivers of several myeloid malignancies, AEL is characterized by different degrees of aberrant erythroid maturation. Recent insights from rationale and unexpected mouse models indicate that the erythroid identity in a significant fraction of AEL is based on the impaired activity of transcriptional master regulators like GATA1. Therapeutic restoring of the GATA1 activity could lead to terminal differentiation of aberrantly accumulated erythroblasts, which may resolve PEL and reduce the cellular burden of AEL. However, more research is needed to dissect the critical molecular mechanisms to translate this strategy into clinically effective therapies.

## Disclosures

The authors have no conflicts of interest to disclose.

## Sources of funding

The work on erythroleukemia by J.S. is supported by the Swiss Cancer Research KFS-3487-08-2014 and KFS-4258-08-2017; the Swiss National Science Foundation (31000A_173224), the Novartis Biomedical Research Foundation, Basel, the San Salvatore Foundation, Lugano (201525), and the Wilhelm-Sander Foundation, Munich (2017-035.1). AF was supported by Ligue Contre le Cancer & Fondation pour la Recherche Médicale. TM is supported by Institut National du Cancer (PLBIO-2018-169), PAIR-Pédiatrie/CONECT-AML (COllaborative Network for Children and Teenagers with Acute Myeloblastic Leukemia: INCa-ARC-LIGUE_11905 and Association Laurette Fugain), Société Française des Cancers de l’Enfant, SIRIC-SOCRATE (INCa-DGOS-INSERM_12551).

## Supplementary Material



## References

[1] Bain BJ. Di Guglielmo and his syndromes. Br J Haematol. 2003;120:939–943. 12648062 10.1046/j.1365-2141.2003.04181.x

[2] Bennett JM Catovsky D Daniel MT. Proposals for the classification of the acute leukaemias. French-American-British (FAB) Co-operative Group. Br J Haematol. 1976;33:451–458. 188440 10.1111/j.1365-2141.1976.tb03563.x

[3] Vardiman JW Thiele J Arber DA. The 2008 revision of the World Health Organization (WHO) classification of myeloid neoplasms and acute leukemia: rationale and important changes. Blood. 2009;114:937–951. 19357394 10.1182/blood-2009-03-209262

[4] Arber DA Orazi A Hasserjian R. The 2016 revision to the World Health Organization classification of myeloid neoplasms and acute leukemia. Blood. 2016;127:2391–2405. 27069254 10.1182/blood-2016-03-643544

[5] Hasserjian RP Zuo Z Garcia C. Acute erythroid leukemia: a reassessment using criteria refined in the 2008 WHO classification. Blood. 2010;115:1985–1992. 20040759 10.1182/blood-2009-09-243964 PMC2942006

[6] Kiossoglou KA Mitus WJ Dameshek W. Chromosomal aberrations in acute leukemia. Blood. 1965;26:610–641. 4221200

[7] Castoldi G Mitus WJ Yam LT. Chromosomal studies in erythroleukemia and chronic erythremic myelosis. Blood. 1968;31:202–215. 5238812

[8] Mazzella FM Kowal-Vern A Shrit MA. Acute erythroleukemia: evaluation of 48 cases with reference to classification, cell proliferation, cytogenetics, and prognosis. Am J Clin Pathol. 1998;110:590–598. 9802343 10.1093/ajcp/110.5.590

[9] Santos FP Faderl S Garcia-Manero G. Adult acute erythroleukemia: an analysis of 91 patients treated at a single institution. Leukemia. 2009;23:2275–2280. 19741728 10.1038/leu.2009.181 PMC4217206

[10] Lessard M Struski S Leymarie V. Cytogenetic study of 75 erythroleukemias. Cancer Genet Cytogenet. 2005;163:113–122. 16337853 10.1016/j.cancergencyto.2005.05.006

[11] Hou HA Chou WC Kuo YY. TP53 mutations in de novo acute myeloid leukemia patients: longitudinal follow-ups show the mutation is stable during disease evolution. Blood Cancer J. 2015;5:e331. 26230955 10.1038/bcj.2015.59 PMC4526785

[12] Wang W Wang SA Medeiros LJ. Pure erythroid leukemia. Am J Hematol. 2017;92:292–296. 28006859 10.1002/ajh.24626

[13] Montalban-Bravo G Benton CB Wang SA. More than 1 TP53 abnormality is a dominant characteristic of pure erythroid leukemia. Blood. 2017;129:2584–2587. 28246192 10.1182/blood-2016-11-749903 PMC5418636

[14] Grossmann V Bacher U Haferlach C. Acute erythroid leukemia (AEL) can be separated into distinct prognostic subsets based on cytogenetic and molecular genetic characteristics. Leukemia. 2013;27:1940–1943. 23648669 10.1038/leu.2013.144

[15] Cervera N Carbuccia N Garnier S. Molecular characterization of acute erythroid leukemia (M6-AML) using targeted next-generation sequencing. Leukemia. 2016;30:966–970. 26202927 10.1038/leu.2015.198

[16] Cervera N Carbuccia N Mozziconacci MJ. Revisiting gene mutations and prognosis of ex-M6a-acute erythroid leukemia with regard to the new WHO classification. Blood Cancer J. 2017;7:e594. 28841205 10.1038/bcj.2017.68 PMC5596380

[17] Ping N Sun A Song Y. Exome sequencing identifies highly recurrent somatic GATA2 and CEBPA mutations in acute erythroid leukemia. Leukemia. 2017;31:195–202. 27389056 10.1038/leu.2016.162

[18] Rose D Haferlach T Schnittger S. Subtype-specific patterns of molecular mutations in acute myeloid leukemia. Leukemia. 2017;31:11–17. 27285584 10.1038/leu.2016.163

[19] Iacobucci I Wen J Meggendorfer M. Genomic subtyping and therapeutic targeting of acute erythroleukemia. Nat Genet. 2019;51:694–704. 30926971 10.1038/s41588-019-0375-1 PMC6828160

[20] Cervera N Lhoumeau AC Adélaïde J. Acute erythroid leukemias have a distinct molecular hierarchy from non-erythroid acute myeloid leukemias. Haematologica. 2020;105:e340–e342. 31601691 10.3324/haematol.2019.231142 PMC7327663

[21] Fagnan A Bagger FO Piqué-Borràs MR. Human erythroleukemia genetics and transcriptomes identify master transcription factors as functional disease drivers. Blood. 2020;136:698–714. 32350520 10.1182/blood.2019003062 PMC8215330

[22] Ware AD Birkness J Duffield AS. Molecular evidence of JAK2 p.V617F mutated pure erythroid leukemia arising from polycythemia vera. Virchows Arch. 2018;473:131–135. 29611054 10.1007/s00428-018-2347-8

[23] Rampal R Ahn J Abdel-Wahab O. Genomic and functional analysis of leukemic transformation of myeloproliferative neoplasms. Proc Natl Acad Sci U S A. 2014;111:E5401–E5410. 25516983 10.1073/pnas.1407792111 PMC4273376

[24] Kreft A Springer E Lipka DB. Wild-type JAK2 secondary acute erythroleukemia developing after JAK2-V617F-mutated primary myelofibrosis. Acta Haematol. 2009;122:36–38. 19713696 10.1159/000235773

[25] Pompetti F Spadano A Sau A. Long-term remission in BCR/ABL-positive AML-M6 patient treated with Imatinib Mesylate. Leuk Res. 2007;31:563–567. 16916543 10.1016/j.leukres.2006.05.021

[26] Zhao W Du Y Ho WT. JAK2V617F and p53 mutations coexist in erythroleukemia and megakaryoblastic leukemic cell lines. Exp Hematol Oncol. 2012;1:15. 23210734 10.1186/2162-3619-1-15 PMC3514099

[27] Steensma DP Bejar R Jaiswal S. Clonal hematopoiesis of indeterminate potential and its distinction from myelodysplastic syndromes. Blood. 2015;126:9–16. 25931582 10.1182/blood-2015-03-631747 PMC4624443

[28] Bowman RL Busque L Levine RL. Clonal hematopoiesis and evolution to hematopoietic malignancies. Cell Stem Cell. 2018;22:157–170. 29395053 10.1016/j.stem.2018.01.011 PMC5804896

[29] Micci F Thorsen J Haugom L. Translocation t(1;16)(p31;q24) rearranging CBFA2T3 is specific for acute erythroid leukemia. Leukemia. 2011;25:1510–1512. 21606959 10.1038/leu.2011.100

[30] Panagopoulos I Micci F Thorsen J. Fusion of ZMYND8 and RELA genes in acute erythroid leukemia. PLoS One. 2013;8:e63663. 23667654 10.1371/journal.pone.0063663 PMC3646816

[31] Micci F Thorsen J Panagopoulos I. High-throughput sequencing identifies an NFIA/CBFA2T3 fusion gene in acute erythroid leukemia with t(1;16)(p31;q24). Leukemia. 2013;27:980–982. 23032695 10.1038/leu.2012.266 PMC3626019

[32] Liu H Guinipero TL Schieffer KM. De novo primary central nervous system pure erythroid leukemia/sarcoma with t(1;16)(p31;q24) NFIA/CBFA2T3 translocation. Haematologica. 2020;105:e194–e197. 31949013 10.3324/haematol.2019.231928 PMC7109754

[33] Linnik Y Pastakia D Dryden I. Primary central nervous system erythroid sarcoma with NFIA-CBFA2T3 translocation: a rare but distinct clinicopathologic entity. Am J Hematol. 2020;95:E299–E301. 32697373 10.1002/ajh.25944

[34] King RL Siaghani PJ Wong K. Novel t(1;8)(p31.3;q21.3) NFIA-RUNX1T1 translocation in an infant erythroblastic sarcoma. Am J Clin Pathol. 2020:aqaa216. [Epub ahead of print]. 33313700 10.1093/ajcp/aqaa216

[35] Chisholm KM Heerema-McKenney AE Choi JK. Acute erythroid leukemia is enriched in NUP98 fusions: a report from the Children’s Oncology Group. Blood Adv. 2020;4:6000–6008. 33284945 10.1182/bloodadvances.2020002712 PMC7724911

[36] Graf T Ade N Beug H. Temperature-sensitive mutant of avian erythroblastosis virus suggests a block of differentiation as mechanism of leukaemogenesis. Nature. 1978;275:496–501. 211440 10.1038/275496a0

[37] Beug H Bauer A Dolznig H. Avian erythropoiesis and erythroleukemia: towards understanding the role of the biomolecules involved. Biochim Biophys Acta. 1996;1288:M35–M47. 9011180 10.1016/s0304-419x(96)00032-7

[38] Rietveld LE Caldenhoven E Stunnenberg HG. Avian erythroleukemia: a model for corepressor function in cancer. Oncogene. 2001;20:3100–3109. 11420726 10.1038/sj.onc.1204335

[39] Ivanov X Mladenov Z Nedyalk S Todorov TG. Experimental investigation into avian leukoses. I. Transmission experiments of certain diseases of the avian leukosis complex in Bulgaria. Bulgarian Acad Sci Bull Inst Pathol Comp Animaux Domestiques. 1962;9:5–36.

[40] Rascle A Ferrand N Gandrillon O. Myb-Ets fusion oncoprotein inhibits thyroid hormone receptor/c-ErbA and retinoic acid receptor functions: a novel mechanism of action for leukemogenic transformation by E26 avian retrovirus. Mol Cell Biol. 1996;16:6338–6351. 8887663 10.1128/mcb.16.11.6338 PMC231636

[41] Blair DG Athanasiou M. Ets and retroviruses—transduction and activation of members of the Ets oncogene family in viral oncogenesis. Oncogene. 2000;19:6472–6481. 11175363 10.1038/sj.onc.1204046

[42] Friend C. Cell-free transmission in adult Swiss mice of a disease having the character of a leukemia. J Exp Med. 1957;105:307–318. 13416470 10.1084/jem.105.4.307 PMC2136697

[43] Orkin SH. Differentiation of murine erythroleukemic (Friend) cells: an in vitro model of erythropoiesis. In Vitro. 1978;14:146–154. 342390 10.1007/BF02618181

[44] Fey F Graffi A. Erythroblasten-Leukämie nach Injektion von Virus der myeloischen Leukämie der Maus. Z Krebsforsch. 1965;67:145–151. 4284025

[45] Siegel BV Weaver WJ Koler RD. Mouse erythroleukemia of viral etiology. Nature. 1964;201:1042–1043. 14191586 10.1038/2011042a0

[46] Ney PA D’Andrea AD. Friend erythroleukemia revisited. Blood. 2000;96:3675–3680. 11090047

[47] Moreau-Gachelin F Tavitian A Tambourin P. Spi-1 is a putative oncogene in virally induced murine erythroleukaemias. Nature. 1988;331:277–280. 2827041 10.1038/331277a0

[48] Longmore GD Lodish HF. An activating mutation in the murine erythropoietin receptor induces erythroleukemia in mice: a cytokine receptor superfamily oncogene. Cell. 1991;67:1089–1102. 1662116 10.1016/0092-8674(91)90286-8

[49] Skoda RC Tsai SF Orkin SH. Expression of c-MYC under the control of GATA-1 regulatory sequences causes erythroleukemia in transgenic mice. J Exp Med. 1995;181:1603–1613. 7722440 10.1084/jem.181.5.1603 PMC2191979

[50] Moreau-Gachelin F Wendling F Molina T. Spi-1/PU.1 transgenic mice develop multistep erythroleukemias. Mol Cell Biol. 1996;16:2453–2463. 8628313 10.1128/mcb.16.5.2453 PMC231234

[51] Trempus CS Ward S Farris G. Association of v-Ha-ras transgene expression with development of erythroleukemia in Tg.AC transgenic mice. Am J Pathol. 1998;153:247–254. 9665485 10.1016/S0002-9440(10)65565-4 PMC1852926

[52] Shimizu R Kuroha T Ohneda O. Leukemogenesis caused by incapacitated GATA-1 function. Mol Cell Biol. 2004;24:10814–10825. 15572684 10.1128/MCB.24.24.10814-10825.2004 PMC533998

[53] Torchia EC Boyd K Rehg JE. EWS/FLI-1 induces rapid onset of myeloid/erythroid leukemia in mice. Mol Cell Biol. 2007;27:7918–7934. 17875932 10.1128/MCB.00099-07 PMC2169157

[54] Salek-Ardakani S Smooha G de Boer J. ERG is a megakaryocytic oncogene. Cancer Res. 2009;69:4665–4673. 19487285 10.1158/0008-5472.CAN-09-0075

[55] Tsuzuki S Seto M. Expansion of functionally defined mouse hematopoietic stem and progenitor cells by a short isoform of RUNX1/AML1. Blood. 2012;119:727–735. 22130803 10.1182/blood-2011-06-362277

[56] Gentner E Vegi NM Mulaw MA. VENTX induces expansion of primitive erythroid cells and contributes to the development of acute myeloid leukemia in mice. Oncotarget. 2016;7:86889–86901. 27888632 10.18632/oncotarget.13563 PMC5349961

[57] Thoene S Mandal T Vegi NM. The ParaHox gene Cdx4 induces acute erythroid leukemia in mice. Blood Adv. 2019;3:3729–3739. 31770439 10.1182/bloodadvances.2019000761 PMC6880902

[58] Kosmider O Denis N Lacout C. Kit-activating mutations cooperate with Spi-1/PU.1 overexpression to promote tumorigenic progression during erythroleukemia in mice. Cancer Cell. 2005;8:467–478. 16338660 10.1016/j.ccr.2005.11.009

[59] Munroe DG Peacock JW Benchimol S. Inactivation of the cellular p53 gene is a common feature of Friend virus-induced erythroleukemia: relationship of inactivation to dominant transforming alleles. Mol Cell Biol. 1990;10:3307–3313. 1694008 10.1128/mcb.10.7.3307-3313.1990 PMC360748

[60] Lavigueur A Bernstein A. p53 transgenic mice: accelerated erythroleukemia induction by Friend virus. Oncogene. 1991;6:2197–2201. 1766668

[61] Li JP D’Andrea AD Lodish HF. Activation of cell growth by binding of Friend spleen focus-forming virus gp55 glycoprotein to the erythropoietin receptor. Nature. 1990;343:762–764. 2154701 10.1038/343762a0

[62] Howard JC Berger L Bani MR. Activation of the erythropoietin gene in the majority of F-MuLV-induced erythroleukemias results in growth factor independence and enhanced tumorigenicity. Oncogene. 1996;12:1405–1415. 8622856

[63] Lachman HM Skoultchi AI. Expression of c-myc changes during differentiation of mouse erythroleukaemia cells. Nature. 1984;310:592–594. 6462247 10.1038/310592a0

[64] Dmitrovsky E Kuehl WM Hollis GF. Expression of a transfected human c-myc oncogene inhibits differentiation of a mouse erythroleukaemia cell line. Nature. 1986;322:748–750. 3528861 10.1038/322748a0

[65] Robert-Lézénès J Meneceur P Ray D. Protooncogene expression in normal, preleukemic, and leukemic murine erythroid cells and its relationship to differentiation and proliferation. Cancer Res. 1988;48:3972–3976. 3164254

[66] Matsuzaki T Aisaki Ki Yamamura Y. Induction of erythroid differentiation by inhibition of Ras/ERK pathway in a friend murine leukemia cell line. Oncogene. 2000;19:1500–1508. 10734309 10.1038/sj.onc.1203461

[67] Ferreira R Ohneda K Yamamoto M. GATA1 function, a paradigm for transcription factors in hematopoiesis. Mol Cell Biol. 2005;25:1215–1227. 15684376 10.1128/MCB.25.4.1215-1227.2005 PMC548021

[68] Takahashi S Onodera K Motohashi H. Arrest in primitive erythroid cell development caused by promoter-specific disruption of the GATA-1 gene. J Biol Chem. 1997;272:12611–12615. 9139715 10.1074/jbc.272.19.12611

[69] Takahashi S Komeno T Suwabe N. Role of GATA-1 in proliferation and differentiation of definitive erythroid and megakaryocytic cells in vivo. Blood. 1998;92:434–442. 9657742

[70] Mukai HY Suzuki M Nagano M. Establishment of erythroleukemic GAK14 cells and characterization of GATA1 N-terminal domain. Genes Cells. 2013;18:886–898. 23890289 10.1111/gtc.12084

[71] Weiss MJ Yu C Orkin SH. Erythroid-cell-specific properties of transcription factor GATA-1 revealed by phenotypic rescue of a gene-targeted cell line. Mol Cell Biol. 1997;17:1642–1651. 9032291 10.1128/mcb.17.3.1642 PMC231889

[72] Nichols KE Crispino JD Poncz M. Familial dyserythropoietic anaemia and thrombocytopenia due to an inherited mutation in GATA1. Nat Genet. 2000;24:266–270. 10700180 10.1038/73480 PMC10576470

[73] Letting DL Rakowski C Weiss MJ. Formation of a tissue-specific histone acetylation pattern by the hematopoietic transcription factor GATA-1. Mol Cell Biol. 2003;23:1334–1340. 12556492 10.1128/MCB.23.4.1334-1340.2003 PMC141148

[74] Welch JJ Watts JA Vakoc CR. Global regulation of erythroid gene expression by transcription factor GATA-1. Blood. 2004;104:3136–3147. 15297311 10.1182/blood-2004-04-1603

[75] Shimizu R Engel JD Yamamoto M. GATA1-related leukaemias. Nat Rev Cancer. 2008;8:279–287. 18354416 10.1038/nrc2348

[76] Beck D Thoms JA Perera D. Genome-wide analysis of transcriptional regulators in human HSPCs reveals a densely interconnected network of coding and noncoding genes. Blood. 2013;122:e12–e22. 23974199 10.1182/blood-2013-03-490425

[77] Loughran SJ Kruse EA Hacking DF. The transcription factor Erg is essential for definitive hematopoiesis and the function of adult hematopoietic stem cells. Nat Immunol. 2008;9:810–819. 18500345 10.1038/ni.1617

[78] Taoudi S Bee T Hilton A. ERG dependence distinguishes developmental control of hematopoietic stem cell maintenance from hematopoietic specification. Genes Dev. 2011;25:251–262. 21245161 10.1101/gad.2009211 PMC3034900

[79] Knudsen KJ Rehn M Hasemann MS. ERG promotes the maintenance of hematopoietic stem cells by restricting their differentiation. Genes Dev. 2015;29:1915–1929. 26385962 10.1101/gad.268409.115 PMC4579349

[80] Wilson NK Foster SD Wang X. Combinatorial transcriptional control in blood stem/progenitor cells: genome-wide analysis of ten major transcriptional regulators. Cell Stem Cell. 2010;7:532–544. 20887958 10.1016/j.stem.2010.07.016

[81] Martens JH. Acute myeloid leukemia: a central role for the ETS factor ERG. Int J Biochem Cell Biol. 2011;43:1413–1416. 21664289 10.1016/j.biocel.2011.05.014

[82] Baldus CD Burmeister T Martus P. High expression of the ETS transcription factor ERG predicts adverse outcome in acute T-lymphoblastic leukemia in adults. J Clin Oncol. 2006;24:4714–4720. 16954520 10.1200/JCO.2006.06.1580

[83] Birger Y Goldberg L Chlon TM. Perturbation of fetal hematopoiesis in a mouse model of Down syndrome’s transient myeloproliferative disorder. Blood. 2013;122:988–998. 23719302 10.1182/blood-2012-10-460998 PMC3739041

[84] Stankiewicz MJ Crispino JD. ETS2 and ERG promote megakaryopoiesis and synergize with alterations in GATA-1 to immortalize hematopoietic progenitor cells. Blood. 2009;113:3337–3347. 19168790 10.1182/blood-2008-08-174813 PMC2665899

[85] Carmichael CL Metcalf D Henley KJ. Hematopoietic overexpression of the transcription factor Erg induces lymphoid and erythro-megakaryocytic leukemia. Proc Natl Acad Sci U S A. 2012;109:15437–15442. 22936051 10.1073/pnas.1213454109 PMC3458381

[86] Tang JZ Carmichael CL Shi W. Transposon mutagenesis reveals cooperation of ETS family transcription factors with signaling pathways in erythro-megakaryocytic leukemia. Proc Natl Acad Sci U S A. 2013;110:6091–6096. 23533276 10.1073/pnas.1304234110 PMC3625293

[87] Lengerke C Daley GQ. Caudal genes in blood development and leukemia. Ann N Y Acad Sci. 2012;1266:47–54. 22901255 10.1111/j.1749-6632.2012.06625.x PMC3431192

[88] Pineault N Helgason CD Lawrence HJ. Differential expression of Hox, Meis1, and Pbx1 genes in primitive cells throughout murine hematopoietic ontogeny. Exp Hematol. 2002;30:49–57. 11823037 10.1016/s0301-472x(01)00757-3

[89] Davidson AJ Ernst P Wang Y. cdx4 mutants fail to specify blood progenitors and can be rescued by multiple hox genes. Nature. 2003;425:300–306. 13679919 10.1038/nature01973

[90] Koo S Huntly BJ Wang Y. Cdx4 is dispensable for murine adult hematopoietic stem cells but promotes MLL-AF9-mediated leukemogenesis. Haematologica. 2010;95:1642–1650. 20494928 10.3324/haematol.2010.023168 PMC2948088

[91] Bansal D Scholl C Fröhling S. Cdx4 dysregulates Hox gene expression and generates acute myeloid leukemia alone and in cooperation with Meis1a in a murine model. Proc Natl Acad Sci U S A. 2006;103:16924–16929. 17068127 10.1073/pnas.0604579103 PMC1636555

[92] Ben-David Y Giddens EB Letwin K. Erythroleukemia induction by Friend murine leukemia virus: insertional activation of a new member of the ets gene family, Fli-1, closely linked to c-ets-1. Genes Dev. 1991;5:908–918. 2044959 10.1101/gad.5.6.908

[93] Li Y Luo H Liu T. The ets transcription factor Fli-1 in development, cancer and disease. Oncogene. 2015;34:2022–2031. 24909161 10.1038/onc.2014.162 PMC5028196

[94] Klemsz MJ Maki RA Papayannopoulou T. Characterization of the ets oncogene family member, fli-1. J Biol Chem. 1993;268:5769–5773. 8449942

[95] Athanasiou M Mavrothalassitis G Sun-Hoffman L. FLI-1 is a suppressor of erythroid differentiation in human hematopoietic cells. Leukemia. 2000;14:439–445. 10720139 10.1038/sj.leu.2401689

[96] Delattre O Zucman J Plougastel B. Gene fusion with an ETS DNA-binding domain caused by chromosome translocation in human tumours. Nature. 1992;359:162–165. 1522903 10.1038/359162a0

[97] Li YJ Zhao X Vecchiarelli-Federico LM. Drug-mediated inhibition of Fli-1 for the treatment of leukemia. Blood Cancer J. 2012;2:e54. 22829238 10.1038/bcj.2011.52 PMC3270256

[98] Liu T Xia L Yao Y. Identification of diterpenoid compounds that interfere with Fli-1 DNA binding to suppress leukemogenesis. Cell Death Dis. 2019;10:117. 30741932 10.1038/s41419-019-1363-1 PMC6370842

[99] Wagner K Zhang P Rosenbauer F. Absence of the transcription factor CCAAT enhancer binding protein alpha results in loss of myeloid identity in bcr/abl-induced malignancy. Proc Natl Acad Sci U S A. 2006;103:6338–6343. 16606850 10.1073/pnas.0508143103 PMC1458879

[100] Tsuruta-Kishino T Koya J Kataoka K. Loss of p53 induces leukemic transformation in a murine model of Jak2 V617F-driven polycythemia vera. Oncogene. 2017;36:3300–3311. 28068330 10.1038/onc.2016.478

[101] Di Genua C Valletta S Buono M. C/EBPα and GATA-2 mutations induce bilineage acute erythroid leukemia through transformation of a neomorphic neutrophil-erythroid progenitor. Cancer Cell. 2020;37:690–704.e8. 32330454 10.1016/j.ccell.2020.03.022 PMC7218711

[102] Pique-Borras MR Otzen Bagger F Filgueira Bezerra M. Transformation mechanisms of the Nfia-ETO2 fusion gene associated with pediatric pure acute erythroleukemia. Paper presented at: 61first ASH Annual Meeting & Exposition. Orlando, FL: Blood. 2020

[103] Iacobucci I Qu C Varotto E. Modeling and targeting of erythroleukemia by hematopoietic genome editing. Blood. 2021 January 19. [Epub ahead of print]. doi: 10.1182/blood.2020009103 10.1182/blood.2020009103 PMC7995291 33512458

[104] Kelly LM Gilliland DG. Genetics of myeloid leukemias. Annu Rev Genomics Hum Genet. 2002;3:179–198. 12194988 10.1146/annurev.genom.3.032802.115046

[105] Suh HC Gooya J Renn K. C/EBPalpha determines hematopoietic cell fate in multipotential progenitor cells by inhibiting erythroid differentiation and inducing myeloid differentiation. Blood. 2006;107:4308–4316. 16469877 10.1182/blood-2005-06-2216 PMC1895788

[106] Abdel-Wahab O Manshouri T Patel J. Genetic analysis of transforming events that convert chronic myeloproliferative neoplasms to leukemias. Cancer Res. 2010;70:447–452. 20068184 10.1158/0008-5472.CAN-09-3783 PMC2947340

[107] Joshi SK Davare MA Druker BJ. Revisiting NTRKs as an emerging oncogene in hematological malignancies. Leukemia. 2019;33:2563–2574. 31551508 10.1038/s41375-019-0576-8 PMC7410820

[108] Taylor J Pavlick D Yoshimi A. Oncogenic TRK fusions are amenable to inhibition in hematologic malignancies. J Clin Invest. 2018;128:3819–3825. 29920189 10.1172/JCI120787 PMC6118587

[109] Cocco E Scaltriti M Drilon A. NTRK fusion-positive cancers and TRK inhibitor therapy. Nat Rev Clin Oncol. 2018;15:731–747. 30333516 10.1038/s41571-018-0113-0 PMC6419506

[110] Starnes LM Sorrentino A Pelosi E. NFI-A directs the fate of hematopoietic progenitors to the erythroid or granulocytic lineage and controls beta-globin and G-CSF receptor expression. Blood. 2009;114:1753–1763. 19542302 10.1182/blood-2008-12-196196

[111] Steinauer N Guo C Zhang J. Emerging roles of MTG16 in cell-fate control of hematopoietic stem cells and cancer. Stem Cells Int. 2017;2017:12. 10.1155/2017/6301385 PMC5735743 29358956

[112] Thirant C Ignacimouttou C Lopez CK. ETO2-GLIS2 hijacks transcriptional complexes to drive cellular identity and self-renewal in pediatric acute megakaryoblastic leukemia. Cancer Cell. 2017;31:452–465. 28292442 10.1016/j.ccell.2017.02.006

[113] Grossmann V Tiacci E Holmes AB. Whole-exome sequencing identifies somatic mutations of BCOR in acute myeloid leukemia with normal karyotype. Blood. 2011;118:6153–6163. 22012066 10.1182/blood-2011-07-365320

[114] Sportoletti P Sorcini D Guzman AG. Bcor deficiency perturbs erythro-megakaryopoiesis and cooperates with Dnmt3a loss in acute erythroid leukemia onset in mice. Leukemia (Advanced Online). 2020 November 06. [Epub ahead of print]. doi: 10.1038/s41375-020-01075-3 10.1038/s41375-020-01075-3 PMC8257496 33159179

[115] Ren J Finney R Ni K. The chromatin remodeling protein Lsh alters nucleosome occupancy at putative enhancers and modulates binding of lineage specific transcription factors. Epigenetics. 2019;14:277–293. 30861354 10.1080/15592294.2019.1582275 PMC6557562

[116] Brumbaugh J Kim IS Ji F. Inducible histone K-to-M mutations are dynamic tools to probe the physiological role of site-specific histone methylation in vitro and in vivo. Nat Cell Biol. 2019;21:1449–1461. 31659274 10.1038/s41556-019-0403-5 PMC6858577

[117] Leonards K Almosailleakh M Tauchmann S. Nuclear interacting SET domain protein 1 inactivation impairs GATA1-regulated erythroid differentiation and causes erythroleukemia. Nat Commun. 2020;11:2807. 32533074 10.1038/s41467-020-16179-8 PMC7293310

[118] Briones V Muegge K. The ghosts in the machine: DNA methylation and the mystery of differentiation. Biochim Biophys Acta. 2012;1819:757–762. 22381140 10.1016/j.bbagrm.2012.02.013 PMC7477944

[119] Sun LQ Lee DW Zhang Q. Growth retardation and premature aging phenotypes in mice with disruption of the SNF2-like gene, PASG. Genes Dev. 2004;18:1035–1046. 15105378 10.1101/gad.1176104 PMC406293

[120] Fan T Schmidtmann A Xi S. DNA hypomethylation caused by Lsh deletion promotes erythroleukemia development. Epigenetics. 2008;3:134–142. 18487951 10.4161/epi.3.3.6252 PMC3113485

[121] Lee DW Zhang K Ning ZQ. Proliferation-associated SNF2-like gene (PASG): a SNF2 family member altered in leukemia. Cancer Res. 2000;60:3612–3622. 10910076

[122] Pastore F Levine RL. Epigenetic regulators and their impact on therapy in acute myeloid leukemia. Haematologica. 2016;101:269–278. 26928248 10.3324/haematol.2015.140822 PMC4815718

[123] Wagner EJ Carpenter PB. Understanding the language of Lys36 methylation at histone H3. Nat Rev Mol Cell Biol. 2012;13:115–126. 22266761 10.1038/nrm3274 PMC3969746

[124] Bennett RL Swaroop A Troche C Licht JD. The role of nuclear receptor-binding SET domain family histone lysine methyltransferases in cancer. Cold Spring Harb Perspect Med. 2017;7:a026708. 28193767 10.1101/cshperspect.a026708 PMC5453381

[125] Hollink IH van den Heuvel-Eibrink MM Arentsen-Peters ST. NUP98/NSD1 characterizes a novel poor prognostic group in acute myeloid leukemia with a distinct HOX gene expression pattern. Blood. 2011;118:3645–3656. 21813447 10.1182/blood-2011-04-346643

[126] Mohammad F Helin K. Oncohistones: drivers of pediatric cancers. Genes Dev. 2017;31:2313–2324. 29352018 10.1101/gad.309013.117 PMC5795778

[127] Lu C Jain SU Hoelper D. Histone H3K36 mutations promote sarcomagenesis through altered histone methylation landscape. Science. 2016;352:844–849. 27174990 10.1126/science.aac7272 PMC4928577

[128] Gutiérrez L Caballero N Fernández-Calleja L. Regulation of GATA1 levels in erythropoiesis. IUBMB Life. 2020;72:89–105. 31769197 10.1002/iub.2192

[129] DeVilbiss AW Tanimura N McIver SC. Navigating transcriptional coregulator ensembles to establish genetic networks: a GATA factor perspective. Curr Top Dev Biol. 2016;118:205–244. 27137658 10.1016/bs.ctdb.2016.01.003

[130] Takeda J Yoshida K Nannya Y. Novel molecular pathogenesis and therapeutic target in acute erythroid leukemia. Paper presented at: Annual Meeting of the American Society of Hematology (ASH); December 7–10, 2019; Orlando: Blood.

[131] Adélaïde J Cervera N Guille A. Gains of EPOR and ERG genes in adult erythroleukaemia. Br J Haematol. 2020;189:e174–e177. 32227335 10.1111/bjh.16586

[132] Garnett C Cruz Hernandez D Vyas P. GATA1 and cooperating mutations in myeloid leukaemia of Down syndrome. IUBMB Life. 2020;72:119–130. 31769932 10.1002/iub.2197

[133] Asai T Liu Y Bae N. The p53 tumor suppressor protein regulates hematopoietic stem cell fate. J Cell Physiol. 2011;226:2215–2221. 21660944 10.1002/jcp.22561 PMC3081536

[134] Prokocimer M Molchadsky A Rotter V. Dysfunctional diversity of p53 proteins in adult acute myeloid leukemia: projections on diagnostic workup and therapy. Blood. 2017;130:699–712. 28607134 10.1182/blood-2017-02-763086 PMC5659817

[135] Schneider RK Schenone M Ferreira MV. Rps14 haploinsufficiency causes a block in erythroid differentiation mediated by S100A8 and S100A9. Nat Med. 2016;22:288–297. 26878232 10.1038/nm.4047 PMC4870050

[136] Le Goff S Boussaid I Floquet C. p53 activation during ribosome biogenesis regulates normal erythroid differentiation. Blood. 2021;137:89–102. 32818241 10.1182/blood.2019003439

[137] Chen S Wang Q Yu H. Mutant p53 drives clonal hematopoiesis through modulating epigenetic pathway. Nat Commun. 2019;10:5649. 31827082 10.1038/s41467-019-13542-2 PMC6906427

[138] Stein Y Rotter V Aloni-Grinstein R. Gain-of-function mutant p53: all the roads lead to tumorigenesis. Int J Mol Sci. 2019;20:6197. 10.3390/ijms20246197 PMC6940767 31817996

[139] Li D Marchenko ND Schulz R. Functional inactivation of endogenous MDM2 and CHIP by HSP90 causes aberrant stabilization of mutant p53 in human cancer cells. Mol Cancer Res. 2011;9:577–588. 21478269 10.1158/1541-7786.MCR-10-0534 PMC3097033

[140] Schulz-Heddergott R Moll UM. Gain-of-function (GOF) mutant p53 as actionable therapeutic target. Cancers (Basel). 2018;10:188. 10.3390/cancers10060188 PMC6025530 29875343

[141] Boettcher S Miller PG Sharma R. A dominant-negative effect drives selection of TP53 missense mutations in myeloid malignancies. Science. 2019;365:599–604. 31395785 10.1126/science.aax3649 PMC7327437

[142] Yamamoto S Iwakuma T. Regulators of oncogenic mutant TP53 gain of function. Cancers (Basel). 2018;11:4. 10.3390/cancers11010004 PMC6356290 30577483

[143] Trainor CD Mas C Archambault P. GATA-1 associates with and inhibits p53. Blood. 2009;114:165–173. 19411634 10.1182/blood-2008-10-180489 PMC2710945

[144] Zhu J Sammons MA Donahue G. Gain-of-function p53 mutants co-opt chromatin pathways to drive cancer growth. Nature. 2015;525:206–211. 26331536 10.1038/nature15251 PMC4568559

[145] Barbosa K Li S Adams PD. The role of TP53 in acute myeloid leukemia: Challenges and opportunities. Genes Chromosomes Cancer. 2019;58:875–888. 31393631 10.1002/gcc.22796 PMC12042961

[146] Krivtsov AV Evans K Gadrey JY. A menin-MLL inhibitor induces specific chromatin changes and eradicates disease in models of MLL-rearranged leukemia. Cancer Cell. 2019;36:660–673.e11. 31821784 10.1016/j.ccell.2019.11.001 PMC7227117

[147] Bernard E Nannya Y Hasserjian RP. Implications of TP53 allelic state for genome stability, clinical presentation and outcomes in myelodysplastic syndromes. Nat Med. 2020;26:1549–1556. 32747829 10.1038/s41591-020-1008-z PMC8381722

[148] Zhang J Kong G Rajagopalan A. p53-/- synergizes with enhanced NrasG12D signaling to transform megakaryocyte-erythroid progenitors in acute myeloid leukemia. Blood. 2017;129:358–370. 27815262 10.1182/blood-2016-06-719237 PMC5248933

[149] Zhao Z Zuber J Diaz-Flores E. p53 loss promotes acute myeloid leukemia by enabling aberrant self-renewal. Genes Dev. 2010;24:1389–1402. 20595231 10.1101/gad.1940710 PMC2895198

[150] Lyko F. The DNA methyltransferase family: a versatile toolkit for epigenetic regulation. Nat Rev Genet. 2018;19:81–92. 29033456 10.1038/nrg.2017.80

[151] Lio CJ Yuita H Rao A. Dysregulation of the TET family of epigenetic regulators in lymphoid and myeloid malignancies. Blood. 2019;134:1487–1497. 31467060 10.1182/blood.2019791475 PMC6839946

[152] Li Z Cai X Cai CL. Deletion of Tet2 in mice leads to dysregulated hematopoietic stem cells and subsequent development of myeloid malignancies. Blood. 2011;118:4509–4518. 21803851 10.1182/blood-2010-12-325241 PMC3952630

[153] Moran-Crusio K Reavie L Shih A. Tet2 loss leads to increased hematopoietic stem cell self-renewal and myeloid transformation. Cancer Cell. 2011;20:11–24. 21723200 10.1016/j.ccr.2011.06.001 PMC3194039

[154] Challen GA Sun D Jeong M. Dnmt3a is essential for hematopoietic stem cell differentiation. Nat Genet. 2011;44:23–31. 22138693 10.1038/ng.1009 PMC3637952

[155] Izzo F Lee SC Poran A. DNA methylation disruption reshapes the hematopoietic differentiation landscape. Nat Genet. 2020;52:378–387. 32203468 10.1038/s41588-020-0595-4 PMC7216752

[156] Yan H Wang Y Qu X. Distinct roles for TET family proteins in regulating human erythropoiesis. Blood. 2017;129:2002–2012. 28167661 10.1182/blood-2016-08-736587 PMC5383871

[157] Qu X Zhang S Wang S. TET2 deficiency leads to stem cell factor-dependent clonal expansion of dysfunctional erythroid progenitors. Blood. 2018;132:2406–2417. 30254129 10.1182/blood-2018-05-853291 PMC6265651

[158] Ketkar S Verdoni AM Smith AM. Remethylation of Dnmt3a-/- hematopoietic cells is associated with partial correction of gene dysregulation and reduced myeloid skewing. Proc Natl Acad Sci U S A. 2020;117:3123–3134. 31996479 10.1073/pnas.1918611117 PMC7022185

[159] Yang L Chen Z Stout ES. Methylation of a CGATA element inhibits binding and regulation by GATA-1. Nat Commun. 2020;11:2560. 32444652 10.1038/s41467-020-16388-1 PMC7244756

[160] Jeong JJ Gu X Nie J. Cytokine-regulated phosphorylation and activation of TET2 by JAK2 in hematopoiesis. Cancer Discov. 2019;9:778–795. 30944118 10.1158/2159-8290.CD-18-1138 PMC6697164

[161] Zhang X Su J Jeong M. DNMT3A and TET2 compete and cooperate to repress lineage-specific transcription factors in hematopoietic stem cells. Nat Genet. 2016;48:1014–1023. 27428748 10.1038/ng.3610 PMC4957136

[162] Castillo-Aguilera O Depreux P Halby L Arimondo PB Goossens L. DNA methylation targeting: the DNMT/HMT crosstalk challenge. Biomolecules. 2017;7:3. 10.3390/biom7010003 PMC5372715 28067760

[163] Almeida AM Prebet T Itzykson R. Clinical outcomes of 217 patients with acute erythroleukemia according to treatment type and line: a retrospective multinational study. Int J Mol Sci. 2017;18:837. 10.3390/ijms18040837 PMC5412421 28420120

[164] Weinberg DN Papillon-Cavanagh S Chen H. The histone mark H3K36me2 recruits DNMT3A and shapes the intergenic DNA methylation landscape. Nature. 2019;573:281–286. 31485078 10.1038/s41586-019-1534-3 PMC6742567

[165] Karia D Gilbert RCG Biasutto AJ. The histone H3K4 demethylase JARID1A directly interacts with haematopoietic transcription factor GATA1 in erythroid cells through its second PHD domain. R Soc Open Sci. 2020;7:191048. 32218938 10.1098/rsos.191048 PMC7029945

[166] Cardin S Bilodeau M Roussy M. Human models of NUP98-KDM5A megakaryocytic leukemia in mice contribute to uncovering new biomarkers and therapeutic vulnerabilities. Blood Adv. 2019;3:3307–3321. 31698461 10.1182/bloodadvances.2019030981 PMC6855103

[167] Ley TJ Miller C Ding L. Genomic and epigenomic landscapes of adult de novo acute myeloid leukemia. N Engl J Med. 2013;368:2059–2074. 23634996 10.1056/NEJMoa1301689 PMC3767041

[168] Yoshida K Toki T Okuno Y. The landscape of somatic mutations in Down syndrome-related myeloid disorders. Nat Genet. 2013;45:1293–1299. 24056718 10.1038/ng.2759

[169] Labuhn M Perkins K Matzk S. Mechanisms of progression of myeloid preleukemia to transformed myeloid leukemia in children with down syndrome. Cancer Cell. 2019;36:340. 31526763 10.1016/j.ccell.2019.08.014

[170] Mazumdar C Shen Y Xavy S. Leukemia-associated cohesin mutants dominantly enforce stem cell programs and impair human hematopoietic progenitor differentiation. Cell Stem Cell. 2015;17:675–688. 26607380 10.1016/j.stem.2015.09.017 PMC4671831

[171] Sasca D Yun H Giotopoulos G. Cohesin-dependent regulation of gene expression during differentiation is lost in cohesin-mutated myeloid malignancies. Blood. 2019;134:2195–2208. 31515253 10.1182/blood.2019001553 PMC7484777

