# Supplementary material for: Molecular Landscapes and Models of Acute Erythroleukemia
Source: Hemasphere. 2021 Apr 21;5(5):e558. doi: 10.1097/HS9.0000000000000558 (PMC8061683; doi:10.1097/HS9.0000000000000558)
Supplement: Supplementary file 1 [file hs9-5-e558-s001.docx]

|  | Grossmann  (14) | Cervera  (15) | Ping  (17) | Rose  (18) | Fagnan (21) | Iacobucci (19) | Montalban-B.  (13) |
| --- | --- | --- | --- | --- | --- | --- | --- |
| **TP53** | 43.5% | 35.0% | 5.2% | 36% | 36.4% | 38.7% | 92% |
| **NPM1** | 16.3% | 17.5% | 15.5% | 19% | 0.0% | 14.5% |  |
| **TET2** | 0.0% | 10.0% | 3.4% | 12% | 24.2% | 13.7% |  |
| **DNMT3A** | 13.0% | 20.0% | 3.4% | 11% | 15.2% | 12.9% |  |
| **RUNX1** | 7.6% | 10.0% | 3.4% | 9% | 21.2% | 12.1% |  |
| **ASXL1** | 7.6% | 2.5% | 3.4% | 7% | 15.2% | 12.1% |  |
| **IDH2** | 4.3% | 15.0% | 0.0% | 5% | 9.1% | 10.5% |  |
| **STAG2** | 0.0% | 5.0% | 0.0% |  | 3.0% | 8.1% |  |
| **KMT2A** | 6.5% | 5.0% | 0.0% |  | 0.0% | 13.7% |  |
| **FLT3** | 3.3% | 5.0% | 1.7% | 6% | 3.0% | 5.6% |  |
|  |  |  |  |  |  |  |  |
| **«Epigenetic»** | 23.9% | 47.5% | 19.0% |  | 45.5% | 72.6% |  |
| **«Signaling»** | 21.7% | 32.5% | 0.0% |  | 42.4% | 50.0% |  |
| **«Transcription»** | 21.7% | 17.5% | 50.0% |  | 42.4% | 50.0% |  |
|  |  |  |  |  |  |  |  |
| **Seq. tool** | **Targeted** | **Targeted** | **WES** | **Targeted** | **WES** | **WES** | **Targeted** |
| **Diagnosis** | AEL | AEL | AEL | AEL/AML | AEL | AEL/PEL | PEL |
|  |  |  |  |  |  |  |  |
| **Patient number** | 92 | 40 | 58 | 142/4373 | 33 | 124 | 12 |

**Fagnan *et al*.**

Suppl. Table 1
